# Therapeutic potential of okra (*Abelmoschus esculentus*) in dysglycaemia and metabolic dysfunction: A systematic review and meta‐analysis across the diabetes spectrum

**DOI:** 10.1113/EP093293

**Published:** 2026-03-13

**Authors:** Ali Jafari, Helia Mardani, Mohammad Amin Karimi, MohammadHossein Sahami Gilan, Helia Hemmat, Fatemeh Shoja, Nooshin Enayati Soofi, Ghazaleh Eslamian

**Affiliations:** ^1^ Student Research Committee, Department of Community Nutrition, Faculty of Nutrition Sciences and Food Technology, National Nutrition and Food Technology Research Institute Shahid Beheshti University of Medical Sciences Tehran Iran; ^2^ Systematic Review and Meta‐analysis Expert Group (SRMEG) Universal Scientific Education and Research Network (USERN) Tehran Iran; ^3^ Students’ Scientific Research Center (SSRC) Tehran University of Medical Sciences Tehran Iran; ^4^ School of Medicine Shahid Beheshti University of Medical Sciences Tehran Iran; ^5^ Department of Nursing, Faculty of Nursing and Midwifery Ilam University of Medical Sciences Ilam Iran; ^6^ Electronic Health and Statistics Surveillance Research Center, SRC Islamic Azad University Tehran Iran; ^7^ Department of Nutrition, SRC Islamic Azad University Tehran Iran; ^8^ School of Health Larestan University of Medical Sciences Larestan Iran; ^9^ Department of Cellular and Molecular Nutrition, Faculty of Nutrition and Food Technology, National Nutrition and Food Technology Research Institute Shahid Beheshti University of Medical Sciences Tehran Iran

**Keywords:** *Abelmoschus esculentus*, glycaemic profile, lipid profile, meta‐analysis, okra

## Abstract

The aim of this systematic review and meta‐analysis was to evaluate comprehensively the therapeutic potential of *Abelmoschus esculentus* (okra) supplementation across the diabetes spectrum of key metabolic risk factors. A search was conducted in PubMed, Scopus, Web of Science, EMBASE and the Cochrane Library, up to 23 July 2025, to identify randomized controlled trials evaluating the effects of okra supplementation on metabolic risk factors in diabetes. Fourteen randomized controlled trials published between 2020 and 2025, including a total of 836 participants, were analysed. Okra supplementation led to significant reductions in 2 h postprandial glucose [weighted mean difference (WMD) = −22.39 mg/dL, 95% confidence interval (CI): −41.39 to −3.38; *P *= 0.021], fasting blood sugar (WMD = −23.66 mg/dL, 95% CI: −34.20 to −13.12; *P* < 0.001), glycosylated haemoglobin (WMD = −0.30%, 95% CI: −0.59 to −0.02; *P* = 0.034), homeostatic model assessment for insulin resistance (WMD = −0.59 units, 95% CI: −1.01 to −0.18; *P* = 0.005), low‐density lipoprotein cholesterol (WMD = −8.55 mg/dL, 95% CI: −14.42 to −2.68; *P *= 0.004) and total cholesterol (WMD = −12.58 mg/dL, 95% CI: −22.78 to −2.37; *P *= 0.016) levels. The certainty of evidence was very low for most outcomes, except for diastolic blood pressure and glycosylated haemoglobin, which were rated as low. Regarding methodological quality, six trials were rated as good, two as fair and six as poor. Okra supplementation might improve glycaemic control and lipid profiles, indicating its potential as a complementary approach in diabetes management. Despite limitations from small and heterogeneous trials, these findings support future research on optimal dosing, safety and personalized applications in metabolic disease management.

## Abstract (highlights)

- **What is the topic of this review?**
  This review comprehensively evaluates the therapeutic potential of okra (*Abelmoschus esculentus*) supplementation across the diabetes spectrum of glycaemic control, lipid metabolism and cardiometabolic risk factors.
- **What advances does it highlight?**
  This review provides the most rigorous synthesis of evidence to date and demonstrates that okra supplementation significantly improves multiple interconnected pathophysiological pathways, including fasting blood glucose, postprandial glucose, glycosylated haemoglobin, insulin resistance, low‐density lipoprotein cholesterol and total cholesterol, with differential responses based on disease phenotype, formulation type and patient characteristics, establishing okra as a promising accessible lifestyle intervention for personalized diabetes management.

## Graphical abstract

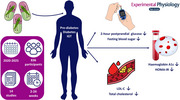

## 1 INTRODUCTION

Cardiovascular disease (CVD) remains the leading cause of mortality worldwide, accounting for an estimated 20.5 million deaths annually, with projections indicating substantial increases driven by population ageing and rising metabolic risk factors (Martin et al., 2024; Vaduganathan et al., 2022). Dysglycaemia, which includes prediabetes and type 2 diabetes mellitus (T2DM), sits at the centre of cardiometabolic pathology, with ∼537 million adults globally affected by diabetes and projections reaching 783 million by 2045 (Cai et al., 2020; ElSayed et al., 2024; Sun et al., 2022). The bidirectional relationship between dysglycaemia and CVD is mediated through endothelial dysfunction, chronic inflammation, oxidative stress, dyslipidaemia and abnormal platelet function, which collectively accelerate atherosclerotic processes and cardiovascular complications (Einarson et al., 2018; Petrie et al., 2018). Despite advances in pharmacological management, the escalating burden of diabetes‐related cardiovascular complications highlights limitations in current therapeutic protocols and drives interest in complementary nutritional interventions (Davies et al., 2022; Zheng et al., 2018). Accumulating evidence suggests that dietary bioactive compounds from plant‐based sources might offer synergistic cardiometabolic benefits through modulation of glucose homeostasis, lipid metabolism, inflammation and oxidative stress pathways (Aune et al., 2016; Buitrago‐Lopez et al., 2011).

Among plant‐derived functional foods, okra (*Abelmoschus esculentus* L., family Malvaceae) has garnered considerable scientific attention for its therapeutic properties across the diabetes spectrum. Okra is exceptionally rich in bioactive constituents, including dietary fibre, mucilaginous polysaccharides, flavonoids (particularly quercetin and catechin derivatives), phenolic compounds, vitamins and essential minerals (Durazzo et al., 2018; Elkhalifa et al., 2021). Recent investigations demonstrate that okra polysaccharides exert antidiabetic effects through multiple mechanisms, including inhibition of α‐glucosidase and α‐amylase activities, enhancement of insulin sensitivity, modulation of antioxidant defence systems and favourable effects on gut microbiota composition (Liao et al., 2019; Zhang et al., 2018). Additionally, the high soluble fibre content has been associated with improved postprandial glycaemic responses, enhanced satiety and beneficial lipid profile effects relevant to cardiovascular risk reduction (Gemede et al., 2015; Reynolds et al., 2020). Although emerging evidence suggests promising antihyperglycaemic and cardiometabolic benefits, the clinical evidence base remains heterogeneous, with variations in study design, intervention protocols, okra preparations, dosing regimens and outcome measures contributing to conflicting results (Jafari et al., 2025).

Previous systematic reviews have examined the effects of okra on glycaemic control and cardiometabolic parameters in dysglycaemia (Bahari et al., 2024; Mokgalaboni et al., 2023); however, opportunities remain to synthesize the evidence comprehensively across broader outcome domains and use advanced analytical approaches to identify optimal therapeutic parameters. Therefore, this systematic review and meta‐analysis was conducted in accordance with PRISMA guidelines to provide a comprehensive evaluation of the therapeutic potential of okra supplementation across the dysglycaemia spectrum. Through an exhaustive and updated search strategy, this study systematically incorporates all eligible randomized controlled trials (RCTs) and expands outcome assessment to encompass glycaemic control, insulin resistance markers, lipid profiles anthropometric parameters, blood pressure, liver and renal function tests, and inflammatory biomarkers in prediabetes, T2DM and metabolic syndrome. Furthermore, this analysis uses advanced statistical approaches, including subgroup analyses stratified by multiple effect modifiers (health status, intervention formulation, dosing regimen, duration and co‐interventions), meta‐regression to identify sources of heterogeneity, and non‐linear dose–response modelling to characterize optimal therapeutic thresholds. By implementing structured quality assessment using the Cochrane risk of bias tool and certainty evaluation via the GRADE framework, this comprehensive synthesis provides evidence‐based guidance for the potential integration of okra supplementation into cardiometabolic disease management strategies while addressing critical knowledge gaps in optimal dosing, intervention duration, safety profile and potential interactions with conventional antidiabetic medications.

## 2 MATERIALS AND METHODS

### 2.1 Protocol and registration

This systematic review and meta‐analysis followed a predefined protocol aligned with the PRISMA statement (Moher et al., 2015). The protocol was registered prospectively in the International Prospective Register of Systematic Reviews (PROSPERO) under registration number CRD420251164139 to promote transparency and methodological rigour. Approval for the conduct of this review was granted by the Ethics Committee of Shahid Beheshti of Medical Sciences (IR.SBMU.RETECH.REC.1404.431).

### 2.2 Search strategy and study selection

We performed a comprehensive literature search for RCTs published before 23 July 2025 across multiple electronic databases, including MEDLINE (via PubMed), EMBASE, Cochrane Library (CENTRAL), Scopus and Web of Science. To broaden coverage, we hand‐searched the reference lists of relevant reviews and publications. Databases such as ProQuest, which mainly host dissertations, theses and selected evidence‐based collections, were excluded because they focus less on primary peer‐reviewed trials and are likely to overlap substantially with the content of the selected databases, contributing few additional relevant trials for this review.

The review targeted the effects of okra supplementation on key indicators of metabolic health among individuals with diabetes, including body mass index (BMI), weight, diastolic blood pressure (DBP), systolic blood pressure (SBP), 2 h PPG, fasting insulin, fasting blood sugar (FBS), haemoglobin A1c (HbA1c), homeostatic model assessment for insulin resistance (HOMA‐IR), high‐sensitivity C‐reactive protein (hs‐CRP), high‐density lipoprotein cholesterol (HDL‐C), low‐density lipoprotein cholesterol (LDL‐C), total cholesterol (TC), triglycerides (TG), alkaline phosphatase (ALP), alanine aminotransferase (ALT), aspartate aminotransferase (AST), blood urea nitrogen (BUN) and creatinine.

Search strings were constructed using terms such as ‘*Abelmoschus esculentus*’, ‘okra’, ‘diabetes mellitus’ and ‘randomized controlled trial’, informed by Medical Subject Headings (MeSH) and Emtree terms. Boolean operators and database‐specific adaptations were applied to optimize retrieval (Table S1).

### 2.3 Inclusion/exclusion criteria and data extraction

Eligible studies were limited to RCTs in parallel or crossover formats that included a comparator arm and were published in English. The research question was framed according to the PICO model: population [adults ≥18 years of age with dysglycaemia across the diabetes spectrum, including prediabetes, impaired glucose tolerance (IGT), gestational diabetes mellitus, type 1 diabetes mellitus, T2DM and other diabetes‐related metabolic conditions); intervention (okra‐based interventions, such as fresh pods, dried powder or extracts); comparator (placebo, usual care or lifestyle change); and outcomes (changes in anthropometric measures, blood pressure, glycaemic profile, inflammatory markers, lipid profile, liver and renal function tests).

Studies were excluded if they used non‐randomized designs, quasi‐ or semi‐experimental designs, involved paediatric populations or focused solely on animal/in vitro models. Also disqualified were narrative reviews, cohort or case–control investigations, unpublished data without full reporting or trials lacking quantifiable pre‐ and postintervention data on targeted outcomes. Interventions combining okra with unrelated agents (e.g., pharmaceuticals) or non‐English publications were also omitted.

Two independent investigators (H.M. and M.K.) screened titles, abstracts and full texts against these criteria, importing results into EndNote X7 to remove duplicates and generate a PRISMA flow diagram. A tailored extraction template was used by two researchers (M.S. and H.H.) to compile details on study authorship, publication date, geographical setting, participant demographics, intervention protocols and end‐point data. Disagreements were addressed through discussion and, if needed, adjudicated by a third reviewer (A.J.).

### 2.4 Methodological quality assessment and evaluation of the strength of evidence

Risk of bias in included RCTs was appraised by two reviewers (A.J. and N.S.) using the Cochrane Risk of Bias 2 (RoB 2) tool, evaluating domains such as randomization processes, deviations from intended interventions, missing outcome data, measurement bias and selective reporting. Conflicts were addressed through deliberation or input from a third assessor (Gh.E.).

The overall certainty of evidence for each outcome was graded using the GRADE approach, categorizing it as high, moderate, low or very low quality. Ratings were informed by initial RCT evidence downgraded for risks including inconsistency, imprecision, indirectness and publication bias, or upgraded for large magnitude of effect or dose–response gradients. Assessments were conducted independently by two evaluators (H.M. and M.K.), with discrepancies mediated by A.J.

### 2.5 Statistical analysis

Analyses were executed in Stata v.15.0 (StataCorp, College Station, TX, USA). We abstracted mean changes, SDs and sample sizes from treatment and control arms for all relevant outcomes. Pooled effects were estimated as weighted mean differences (WMDs) to accommodate varying measurement scales.

A random‐effects model using the DerSimonian–Laird approach was used to account for between‐study variability (DerSimonian & Laird, 1986). For studies reporting only end‐point SDs, change‐score SDs were imputed via the Cochrane formula, assuming a correlation of 0.5 between baseline and follow‐up. Standard errors (SEs) were converted to SDs as SD = SE × √*n*, where *n* is group size.

Heterogeneity was quantified using the *I* ^2^ statistic, supplemented by 95% prediction intervals to contextualize variability. Subgroup explorations examined modifiers such as heath condition (individual with diabetes vs. individual with IGT or prediabetes), intervention approach (standardized okra intervention vs. dietary and lifestyle management approaches), intervention type (capsules and tablets vs. liquid and natural forms), treatment pairing protocol (pharmaceutical or dietary co‐treatments vs. solo treatment approaches), control strategy design (placebo control methodology vs. standard care protocol), okra dosage (<4000vs. ≥4000 mg/day), intervention duration (≤8 vs. >8 weeks), intervention age (<55 vs. ≥55 years), country (Iran vs. other countries), baseline BMI [healthy weight or overweight (<30 kg/m^2^) vs. obese (≥30 kg/m^2^)], sample size (≤60 vs. >60 participants), publication year (before and including 2022 vs. after 2022) and study quality (poor or fair vs. good). Meta‐regression analyses were performed to assess the effects of okra dosage and intervention duration on the study outcomes (Orsini et al., 2006). Moreover, a non‐linear dose–response model was used to explore the relationship between varying supplementation levels and the corresponding health effects, aiming to provide evidence‐based insights for determining the optimal dose and intervention duration (Xu & Doi, 2018). Publication bias was assessed through funnel plot inspection and Egger's test (Egger et al., 1997).

## 3 RESULTS

### 3.1 Study selection

**FIGURE 1:**
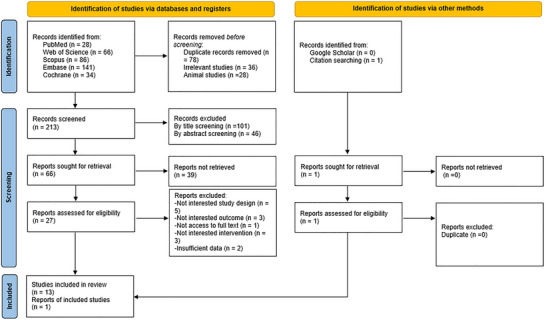
Flowchart of study selection for inclusion trials in the systematic review.

A comprehensive search was performed in PubMed (*n* = 28), Web of Science (*n* = 66), Scopus (*n* = 86), Embase (*n* = 141) and the Cochrane Library (*n* = 34), resulting in a total of 355 records. After removal of duplicates (*n* = 78), animal studies (*n* = 28) and non‐relevant publications (*n* = 36), 213 studies remained for title and abstract screening. Of these, 147 were excluded owing to irrelevant titles (*n* = 101) or irrelevant abstracts (*n* = 46). Subsequently, 66 articles were retrieved for full‐text assessment; however, 39 could not be obtained, leaving 27 studies for eligibility assessment. During this stage, 14 studies were excluded for reasons such as unsuitable study design (*n* = 5), irrelevant outcomes (*n* = 3), insufficient data (*n* = 2), mismatched intervention (*n* = 3) and no access to the full text (*n* = 1) (Table S2). Finally, 14 studies were included in the meta‐analysis, with 13 identified through database searches and one via manual searching. The selection process followed PRISMA guidelines (Figure 1).

### 3.2 Study characteristics

The characteristics of the included studies are summarized in Table 1. The WMDs and 95% confidence intervals (CI) for changes in BMI, WC, weight, DBP, SBP, 2 h PPG, fasting insulin, FBS, HbA1c, HOMA‐IR, hs‐CRP, HDL‐C, LDL‐C, TC, TG, ALP, ALT, AST, BUN and creatinine are presented in Figures S1–S7. Additionally, funnel plots for evaluating publication bias are presented in Figure S8.

**TABLE 1:** Characteristics of studies included in the meta‐analysis.

Reference	Year	Country	Study design	Health status	Total sample size (sex)	Sample size INT/CON	Dose of supplement (mg/day)	Duration (weeks)	Mean BMI (kg/m^2^) INT/CON	Mean age (years) INT/CON	Type of supplement INT vs. CON	Outcomes	Additional information
Gomathi et al. (2020)	2020	India	R, pilot	T2DM	10 (♀♂)	5/5	50 000	12	26.9 ± 1.06/25.66 ± 5.26	40–60/40–60	Okra juice + metformin/usual care (metformin)	BMI, DBP, SBP, FBS and HbA1c	Fresh okra (50 g) soaked overnight in 200 mL water to extract mucilaginous water
Khodija et al. (2020)	2020	Indonesia	R, controlled trial	T2DM with hypercholesterolaemia	40 (♀♂)	12 + 12/16	40 000	2	17–27/17–27	45–65/45–65	Boiled or steamed okra/usual care	FBS	–
Moradi et al. (2020)	2020	Iran	R, PC, DB	T2DM	48 (♀♂)	25/23	10 000	8	24.90 ± 3.94/25.65 ± 3.46	54.26 ± 7.62/53.33 ± 7.35	Okra powder/placebo (consumable colour)	Weight, BMI, DBP, SBP, fasting insulin, FBS, HbA1c, HDL‐C, HOMA‐IR, LDL‐C, TC and TG	Participants were instructed to consume okra powder blended in yogurt or yogurt alone with dinner and lunch‐time meals
Zhao et al. (2020)	2020	China	R, controlled trial	T2DM with non‐proliferative retinopathy	77 (♀♂)	38/39	5400	24	25.9 ± 3.6/26.4 ± 3.3	59 ± 9.22/56 ± 9.99	Semi‐extractable okra tablets/usual care	ALT, AST, BUN, DBP, FBS, HbA1c, HDL‐C, LDL‐C, SBP, Creatinine, TC and TG	Both groups were under basic treatments and monitoring of relevant indicators according to ‘China Guideline for Type 2 Diabetes’
Nikpayam et al. (2022)	2022	Iran	R, PC, TB	Diabetic nephropathy	55 (♀♂)	30/25	80	10	30.35 ± 5.05/28.64 ± 3.17	62 ± 7/ 61.6 ± 8.5	Capsule: Dried okra extract/placebo	BMI, WC and weight	–
Saatchi et al. (2022)	2022	Iran	R, PC, DB	T2DM	99 (♀♂)	50/49	4000	8	30.2 ± 4.3/31.1 ± 4.1	57.7 ± 9.7/58.3 ± 9.2	Capsule: Okra + oral hypoglycaemic medication/placebo + oral hypoglycaemic medication	BMI, ALT, AST, FBS, DBP, SBP, HDL‐C, LDL‐C, HbA1c, TG, TC and WC	–
Chen et al. (2023)	2023	China	R, PC	IGT	60 (♀♂)	30/30	20 000	8	NR	40.8 ± 5.4/41.4 ± 5.9	Okra powder + lifestyle change/lifestyle change	2 h PPG, ALT, creatinine, FBS, fasting insulin, HbA1c, HDL‐C, HOMA‐IR, LDL‐C, TC and TG	–
Salarfard et al. (2023)	2023	Iran	R, NB, controlled study	Gestational diabetes mellitus	60 (♀)	30/30	6000	4	27.3 ± 4.2/ 26.2 ± 4.0	29.0 ± 3.9/28.0 ± 4.6	Sachet: Okra powder/usual care	2 h PPG and FBS	Were educated on managing their condition through diet and regular monitoring of FBS and 2 h PP glucose using a glucometer
Tavakolizadeh et al. (2023)	2023	Iran	R, PC, DB	T2DM	94 (♀♂)	48/46	3000	12	28.6 ± 2.05/ 29.5 ± 3.36	53.8 ± 3.7/52.8 ± 4.6	Capsule: Powdered okra fruit/placebo	ALP, ALT, AST, BUN, creatinine, DBP, FBS, HbA1c, HDL‐C, HOMA‐IR, hs‐CRP, fasting insulin, LDL‐C, SBP, TC and TG	–
Afsharmanesh et al. (2024)	2024	Iran	R, PC, DB	Prediabetic	70 (♀♂)	35/ 35	3000	8	NR	45.81 ± 6.59/45.61 ± 7.80	Capsule: Okra powder/placebo	ALP, ALT, AST, BUN, Creatinine, HDL‐C, LDL‐C, TC and TG	–
Bahreini et al. (2024)	2024	Iran	R, PC, TB	DN	55 (♀♂)	30/25	80	10	≥25/≥25	62.00 ± 7.00/64.6 ± 8.5	Capsule: DOE/placebo	DBP, HDL‐C, LDL‐C, SBP, TC, TG and weight	–
Nikpayam et al. (2024)	2024	Iran	R, PC, TB	DN	55 (♀♂)	30/25	80	10	30.35 ± 5·05/28.64 ± 3.17	62.00 ± 7.00/61.60 ± 8.50	Capsule: DOE/placebo (carboxymethyl cellulose)	FBS, HbA1c, HOMA‐IR, hs‐CRP and fasting insulin	–
Hesamzadeh et al. (2025)	2025	Iran	R, PC, DB	T2DM	53 (♀♂)	27/26	3000	8	29.96 ± 5.69/29.85 ± 5.05	53.41 ± 6.356/52.73 ± 8.693	Capsule: Dried okra fruit powder/placebo (microcrystalline cellulose)	BMI, FBS, BUN, creatinine, TG, AST, ALT, fasting insulin, HbA1c and HOMA‐IR	Both groups followed their existing non‐insulin oral medications
Raiesifar et al. (2025)	2025	Iran	R, DB	T2DM	60 (♀♂)	30/30	80 000	4	NR	18 ≤/18 ≤	Liquid extract: Okra/usual care (routine diabetic programme)	FBS, 2 h PPG, TG, TC, HDL‐C, LDL‐C and HbA1c	Both groups were under a diabetic nutrition programme with ≥20 g of oral fibre consumed 1000 mg/day metformin

Abbreviations: ♀, female; ♂, male; 2 h PPG, 2 h postprandial glucose; ALP, alkaline phosphatase; ALT, alanine aminotransferase; AST, aspartate aminotransferase; BMI, body mass index; BUN, blood urea nitrogen; CON, control group; DB, double blind; DBP, diastolic blood pressure; DOE, dried okra extract; FBS, fasting blood sugar; HDL‐C, high‐density lipoprotein cholesterol; HOMA‐IR, homeostatic model assessment for insulin resistance; hs‐CRP, high‐sensitivity C‐reactive protein; IGT, impaired glucose tolerance; INT, intervention group; LDL‐C, low‐density lipoprotein cholesterol; NB, non‐blinded; PC, placebo controlled; R, randomized; SBP, systolic blood pressure; TB, triple blind; TC, total cholesterol; TG, triglycerides; WC, waist circumference.

The included studies, published between 2020 and 2025, encompassed a total of 836 participants, with 432 in intervention groups and 404 in control groups. Studies were conducted across four countries: India (Gomathi et al., 2020); Indonesia (Khodija et al., 2020); Iran (Afsharmanesh et al., 2024; Bahreini et al., 2024; Hesamzadeh et al., 2025; Moradi et al., 2020; Nikpayam et al., 2022; Nikpayam et al., 2024; Raiesifar et al., 2025; Saatchi et al., 2022; Salarfard et al., 2023; Tavakolizadeh et al., 2023); and China (Chen et al., 2023; Zhao et al., 2020). All were randomized in design. Sample sizes ranged from 10 to 99 participants. Most trials focused on both sexes (Afsharmanesh et al., 2024; Bahreini et al., 2024; Chen et al., 2023; Gomathi et al., 2020; Hesamzadeh et al., 2025; Khodija et al., 2020; Moradi et al., 2020; Nikpayam et al., 2022; Nikpayam et al., 2024; Raiesifar et al., 2025; Saatchi et al., 2022; Tavakolizadeh et al., 2023; Zhao et al., 2020), whereas one included only females (Salarfard et al., 2023). The intervention protocols varied widely, with okra supplementation administered in doses between 80 and 80 000 mg/day over treatment periods lasting from 2 to 24 weeks.

The number of participants in the intervention and control groups, stratified by outcome, was as follows: BMI, *n* = 359 (intervention, 185; control, 174); WC, *n* = 154 (intervention, 80; control, 74); weight, *n* = 158 (intervention, 85; control, 73); DBP, *n* = 383 (intervention, 196; control, 187); SBP, *n* = 383 (intervention, 196; control, 187); 2 h PPG, *n* = 180 (intervention, 90; control, 90); fasting insulin, *n* = 310 (intervention, 160; control, 150); FBS, *n* = 656 (intervention, 337; control, 319); HbA1c, *n* = 556 (intervention, 283; control, 273); HOMA‐IR, *n* = 310 (intervention, 160; control, 150); hs‐CRP, *n* = 149 (intervention, 78; control, 71); HDL‐C, *n* = 563 (intervention, 286; control, 277); LDL‐C, *n* = 563 (intervention, 286; control, 277); TC, *n* = 563 (intervention, 286; control, 277); TG, *n* = 616 (intervention, 313; control, 303); ALP, *n* = 164 (intervention, 83; control, 81); ALT, *n* = 453 (intervention, 228; control, 225); AST, *n* = 393 (intervention, 198; control, 195); BUN, *n* = 294 (intervention, 148; control, 146); and creatinine, *n* = 354 (intervention, 178; control, 176).

### 3.3 Qualitative data assessment

The quality of the 14 studies included in this analysis was evaluated using the Cochrane risk of bias tool (Table 2). Of these, six studies (Bahreini et al., 2024; Moradi et al., 2020; Nikpayam et al., 2022, 2024; Raiesifar et al., 2025; Tavakolizadeh et al., 2023) were classified as good, two (Hesamzadeh et al., 2025; Zhao et al., 2020) as fair and six (Afsharmanesh et al., 2024; Chen et al., 2023; Gomathi et al., 2020; Khodija et al., 2020; Saatchi et al., 2022; Salarfard et al., 2023) as poor.

**TABLE 2:** Quality of included studies in the meta‐analysis.

Reference	Random sequence generation	Allocation concealment	Blinding of participants and personnel	Blinding of outcome assessment	Incomplete outcome data	Selective outcome reporting	Other sources of bias	Overall quality
Gomathi et al. (2020)	U	H	H	H	U	L	L	Poor
Khodija et al. (2020)	U	H	H	H	H	L	L	Poor
Moradi et al. (2020)	L	L	L	L	L	L	L	Good
Zhao et al. (2020)	L	H	L	L	L	L	L	Fair
Nikpayam et al. (2022)	L	L	L	L	L	L	L	Good
Saatchi et al. (2022)	L	L	L	H	H	L	L	Poor
Chen et al. (2023)	L	H	H	H	H	L	L	Poor
Salarfard et al. (2023)	U	H	H	H	H	L	L	Poor
Tavakolizadeh et al. (2023)	L	L	L	L	L	L	L	Good
Afsharmanesh et al. (2024)	L	L	L	H	H	L	L	Poor
Bahreini et al. (2024)	L	L	L	L	L	L	L	Good
Nikpayam et al. (2024)	L	L	L	L	L	L	L	Good
Hesamzadeh et al. (2025)	L	L	L	L	H	L	L	Fair
Raiesifar et al. (2025)	L	L	L	L	L	L	L	Good

Abbreviations: H, high risk of bias; L, low risk of bias; U, unclear risk of bias.

### 3.4 Effects of okra supplementation on anthropometric indices

Okra supplementation did not show significant effects on BMI (WMD = −0.21 kg/m^2^, 95% CI: −0.70 to 0.27; *P* = 0.389; Figure S1), WC (WMD = 0.40 cm, 95% CI: −1.56 to 2.35; *P* = 0.691) or weight (WMD = −0.12 kg, 95% CI: −2.44 to 2.19; *P* = 0.918). Egger's test indicated a significant publication bias for BMI (*P* = 0.006). Sensitivity analyses, excluding individual studies, revealed no changes in the reported results.

### 3.5 Effects of okra supplementation on blood pressure

Okra supplementation did not demonstrate significant effects on DBP (WMD = −0.70 mmHg, 95% CI: −1.86 to 0.46; *P* = 0.234; Figure S2) or SBP (WMD = −1.08 mmHg, 95% CI: −3.61 to 1.45; *P* = 0.404). Egger's test indicated no significant publication bias for DBP (*P* = 0.488) or SBP (*P* = 0.718). Sensitivity analyses did not reveal any single study that significantly altered the overall effect estimates for DBP or SBP.

### 3.6 Effects of okra supplementation on glycaemic profile

Our meta‐analysis showed that okra supplementation resulted in a significant reduction in 2 h PPG (WMD = −22.39 mg/dL, 95% CI: −41.39 to −3.38; *P *= 0.021; Figure S3), FBS (WMD = −23.66 mg/dL, 95% CI: −34.20 to −13.12; *P* < 0.001), HbA1c (WMD = −0.30%, 95% CI: −0.59 to −0.02; *P* = 0.034) and HOMA‐IR (WMD = −0.59 units, 95% CI: −1.01 to −0.18; *P* = 0.005). No significant effect was observed on fasting insulin (WMD = 0.65 µU/mL, 95% CI: −0.73 to 2.04; *P* = 0.356). No significant publication bias was observed for these glycaemic parameters via Egger's test, including 2 h PPG (*P* = 0.954), fasting insulin (*P* = 0.821), FBS (*P* = 0.271), HbA1c (*P* = 0.798) and HOMA‐IR (*P* = 0.791).

Sensitivity analysis showed that the overall effect on 2 h PPG became non‐significant after removal of the study by Chen et al. (2023) (WMD: −35.19 mg/dL; 95% CI: −70.41 to 0.01), Raiesifar et al. (2025) (WMD: −9.49 mg/dL; 95% CI: −27.21 to 8.23) or Salarfard et al. (2023) (WMD: −26.59 mg/dL; 95% CI: −79.58 to 26.40). Likewise, the significance for HbA1c was lost after removal of the studies conducted by Nikpayam et al. (2022) (WMD: −0.29%; 95% CI: −0.60 to 0.01), Tavakolizadeh et al. (2023) (WMD: −0.24%; 95% CI: −0.54 to 0.06), Gomathi et al. (2020) (WMD: −0.23%; 95% CI: −0.47 to 0.02), Raiesifar et al. (2025) (WMD: −0.33%; 95% CI: −0.72 to 0.05) or Saatchi et al. (2022) (WMD: −0.26%; 95% CI: −0.58 to 0.06). The effect on HOMA‐IR also became non‐significant upon removal of the study by Chen et al. (2023) (WMD: −0.51 units; 95% CI: −1.16 to 0.13).

### 3.7 Effects of okra supplementation on inflammatory markers

The analysis of hs‐CRP showed no significant overall reduction with okra administration (WMD = −1.21 µU/mL, 95% CI: −3.24 to 0.81; *P *= 0.239; Figure S4). No publication bias was suggested. However, the sensitivity analysis showed a significant reduction in hs‐CRP upon exclusion of the study by Nikpayam et al. (2022), which resulted in a WMD of −2.30 µU/mL (95% CI: −3.54 to −1.06).

### 3.8 Effects of okra supplementation on lipid profile

According to our results, okra supplementation significantly reduced LDL‐C (WMD = −8.55 mg/dL, 95% CI: −14.42 to −2.68; *P *= 0.004; Figure S5) and TC (WMD = −12.58 mg/dL, 95% CI: −22.78 to −2.37; *P *= 0.016). No significant effects were found for HDL‐C (WMD = 3.01 mg/dL, 95% CI: −0.57 to 6.59; *P *= 0.099) or TG (WMD = −10.74 mg/dL, 95% CI: −26.69 to 5.22; *P* = 0.187). Egger's test indicated a significant publication bias for TG (*P* = 0.013), whereas no significant publication bias was observed for HDL‐C (*P *= 0.653), LDL‐C (*P* = 0.064) or TC (*P* = 0.109).

Sensitivity analysis showed that the effect on HDL‐C became significant upon removal of the study by Saatchi et al. (2022) (WMD: 3.84 mg/dL, 95% CI: 0.00 to 7.67). The significance of TC was lost upon removal of the studies by Afsharmanesh et al. (2024) (WMD: −11.67 mg/dL, 95% CI: −23.86 to 0.52) and Tavakolizadeh et al. (2023) (WMD: −11.14 mg/dL, 95% CI: −22.81 to 0.53). The effect on TG became significant with removal of the study by Saatchi et al. (2022) (WMD: −14.90 mg/dL, 95% CI: −28.68 to −1.12).

### 3.9 Effects of okra supplementation on liver function tests

Okra supplementation did not show significant effects on ALP (WMD = 2.73 IU/L, 95% CI: −2.41 to 7.87; *P* = 0.298; Figure S6), ALT (WMD = −1.37 IU/L, 95% CI: −4.42 to 1.68; *P* = 0.380), AST (WMD = −1.51 IU/L, 95% CI: −3.61 to 0.59; *P* = 0.159), BUN (WMD = 0.24 mg/dL, 95% CI: −0.39 to 0.87; *P* = 0.458) or creatinine (WMD = −0.01 mg/dL, 95% CI: −0.04 to 0.01; *P* = 0.169). Egger's test suggested no publication bias among the parameters in this group, namely ALT (*P* = 0.965) or AST (*P* = 0.397). However, sensitivity analysis showed that removal of the study by Hesamzadeh et al. (2025) resulted in a significant reduction in AST (WMD: −2.22 IU/L, 95% CI: −4.41 to −0.03).

### 3.10 Effects of okra supplementation on renal function tests

The analysis of BUN did not show significant effects on BUN (WMD = 0.24 mg/dL, 95% CI: −0.39 to 0.87; *P* = 0.458; Figure S7) or creatinine (WMD = −0.01 mg/dL, 95% CI: −0.04 to 0.01; *P* = 0.169). Moreover, based on Egger's test no publication bias was detected for BUN (*P* = 0.149) or creatinine (*P* = 0.156).

### 3.11 Subgroup analysis

When stratified by health condition, insulin, HOMA‐IR, HDL‐C, LDL‐C, TC and AST demonstrated significant alterations among participants with IGT or prediabetes. In contrast, HbA1c showed significant effects only in individuals with diabetes, while FBS was significantly changed in both groups (Table 3).

**TABLE 3:** Description of the analysis and subgroup results of okra on diabetes.

	Studies (*n*)	Participants (*n*)	WMD (95% CI)	*P*−value	Heterogeneity		
					*P* heterogeneity	*I* ^2^	*P* between subgroups
**Analysis and subgroup results of okra supplementation on BMI**							
**Overall effect**	6	359	−0.21 (−0.70, 0.27)	0.389	0.916	0.0%	
**Health condition**							
Individual with diabetes	6	359	−0.21 (−0.70, 0.27)	0.389	0.916	0.0%	
Individual with impaired glucose tolerance or prediabetes	0	0	–	–	–	–	
**Intervention approach**							
Standardized okra interventions	2	101	−0.05 (−1.11, 1.00)	0.922	0.760	0.0%	0.737
Dietary and lifestyle management approaches	4	258	−0.26 (−0.81, 0.29)	0.357	0.736	0.0%	
**Intervention type**							
Capsules and tablets	4	301	−0.26 (−0.79, 0.28)	0.347	0.833	0.0%	0.706
Liquid and natural forms	2	58	−0.002 (−1.20, 1.20)	0.997	0.495	0.0%	
**Treatment pairing protocol**							
Pharmaceutical or dietary co‐treatments	3	200	−0.05 (−0.80, 0.69)	0.892	0.954	0.0%	0.572
Solo treatment approaches	3	159	−0.34 (−0.98, 0.31)	0.307	0.588	0.0	
**Control strategy design**							
Placebo control methodology	5	349	−0.24 (−0.74, 0.25)	0.333	0.927	0.0%	0.441
Standard care protocol	1	10	1.05 (−2.20, 4.30)	0.527	–	–	
**Okra dosage (mg/day)**							
<4000	3	202	−0.33 (0.95, 0.29)	0.300	0.716	0.0%	0.561
≥4000	3	157	−0.03 (−0.82, 0.76)	0.942	0.791	0.0	
**Intervention duration (weeks)**							
≤8	3	200	−0.05 (−0.80, 0.69)	0.892	0.954	0.0%	0.572
>8	3	159	−0.34 (−0.98, 0.31)	0.307	0.588	0.0	
**Intervention age (years)**							
<55	4	205	−0.30 (−0.91, 0.30)	0.324	0.744	0.0%	0.624
≥55	2	154	−0.05 (−0.88, 0.79)	0.913	0.991	0.0	
**Country**							
Iran	5	349	−0.24 (−0.74, 0.25)	0.333	0.927	0.0%	0.441
Other countries	1	10	1.05 (−2.20, 4.30)	0.527	–	–	
**Baseline BMI**							
Healthy weight or overweight (>30 kg/m^2^)	4	205	−0.30 (−0.91, 0.30)	0.324	0.744	0.0%	0.624
Obese (≥30 kg/m^2^)	2	154	−0.05 (−0.88, 0.79)	0.913	0.991	0.0	
Not reported	0	0	–	–	–	–	
**Sample size**							
≤60	4	166	0.02 (−0.79, 0.83)	0.962	0.918	0.0%	0.477
>60	2	193	−0.35 (−0.96, 0.26)	0.264	0.494	0.0	
**Publication year**							
≤2022	4	212	−0.03 (−0.72, 0.65)	0.927	0.925	0.0%	0.457
>2022	2	147	−0.40 (−1.10, 0.29)	0.256	0.501	0.0	
**Study quality**							
Poor or fair	2	109	0.05 (−0.95, 1.05)	0.916	0.528	0.0%	0.547
Good	4	250	−0.30 (−0.86, 0.26)	0.295	0.870	0.0	
**Analysis and subgroup results of okra supplementation on WC**							
**Overall effect**	2	154	0.40 (−1.56, 2.35)	0.691	0.886	0.0%	
**Analysis and subgroup results of** okra supplementation on weight							
**Overall effect**	3	158	−0.12 (−2.44, 2.19)	0.918	0.976	0.0%	
**Analysis and subgroup results of** okra **supplemetation on DBP**							
**Overall effect**	6	383	−0.70 (−1.86, 0.46)	0.234	0.901	0.0%	
**Health condition**							
Individual with diabetes	6	383	−0.70 (−1.86, 0.46)	0.234	0.901	0.0%	
Individual with impaired glucose tolerance or prediabetes	0	0	–	–	–	–	
**Intervention approach**							
Standardized okra interventions	1	48	−0.45 (−3.12, 2.22)	0.741	–	–	0.836
Dietary and lifestyle management approaches	5	335	−0.76 (−2.05, 0.53)	0.246	0.816	0.0	
**Intervention type**							
Capsules and tablets	4	325	−0.78 (−2.09, 0.52)	0.239	0.677	0.0%	0.794
Liquid and natural forms	2	58	−0.41 (−2.93, 2.12)	0.754	0.916	0.0	
**Treatment pairing protocol**							
Pharmaceutical or dietary co‐treatments	2	147	−1.02 (−3.10, 1.05)	0.334	0.503	0.0%	0.716
Solo treatment approaches	4	236	−0.56 (−1.96, 0.84)	0.434	0.797	0.0	
**Control strategy design**							
Placebo control methodology	4	296	−0.90 (−2.21, 0.41)	0.179	0.751	0.0%	0.530
Standard care protocol	2	87	0.00 (−2.48, 2.48)	1.000	1.000	–	
**Okra dosage (mg/day)**							
<4000	2	149	−0.82 (−2.51, 0.87)	0.344	0.391	0.0%	0.857
≥4000	4	234	−0.60 (−2.19, 0.99)	0.458	0.842	0.0	
**Intervention duration (weeks)**							
≤8	2	147	−1.02 (−3.10, 1.05)	0.334	0.503	0%	0.716
>8	4	236	−0.56 (−1.96, 0.84)	0.434	0.797	0	
**Intervention age (years)**							
<55	3	152	−0.43 (−1.95, 1.08)	0.574	0.994	0.0%	0.587
≥55	3	231	−1.08 (−2.89, 0.71)	0.238	0.524	0.0	
**Country**							
Iran	4	296	−0.90 (−2.21, 0.41)	0.179	0.751	0.0%	0.530
Other countries	2	87	0.00 (−2.48, 2.48)	1.000	1.000	–	
**Baseline BMI**							
Healthy weight or overweight (>30 kg/m^2^)	4	229	−0.33 (−1.63, 0.98)	0.627	0.993	0.0%	0.223
Obese (≥30 kg/m^2^)	2	154	−2.08 (−4.57, 0.41)	0.103	0.873	0.0	
Not reported	0	0	–	–	–	–	
**Sample size**							
≤60	3	113	−0.99 (−3.09, 1.12)	0.358	0.712	0.0%	0.752
>60	3	270	−0.58 (−1.97, 0.81)	0.413	0.663	0.0	
**Publication year**							
≤2022	4	234	−0.60 (−2.19, 0.99)	0.458	0.842	0.0%	0.857
>2022	2	149	−0.82 (−2.51, 0.87)	0.344	0.391	0.0	
**Study quality**							
Poor or fair	3	186	−0.69 (−2.67, 1.30)	0.497	0.666	0.0%	0.985
Good	3	197	−0.71 (−2.14, 0.72)	0.329	0.675	0.0	
**Analysis and subgroup results of okra supplementation on SBP**							
**Overall effect**	6	383	−1.08 (−3.61, 1.45)	0.404	0.024	61.4%	
**Health condition**							
Individual with diabetes	6	383	−1.08 (−3.61, 1.45)	0.404	0.024	61.4%	
Individual with impaired glucose tolerance or prediabetes	0	0	–	–	–	–	
**Intervention approach**							
Standardized okra interventions	2	125	−2.06 (−4.37, 0.26)	0.082	0.333	0.0%	0.577
Dietary and lifestyle management approaches	4	258	−0.72 (−4.80, 3.37)	0.730	0.016	70.8	
**Intervention type**							
Capsules and tablets	4	325	−1.54 (−5.00, 1.91)	0.381	0.006	76.2%	0.632
Liquid and natural forms	2	58	−0.36 (−3.77, 3.06)	0.839	0.621	0.0	
**Treatment pairing protocol**							
Pharmaceutical or dietary co‐treatments	2	147	1.25 (−2.35, 4.84)	0.496	0.144	53.2%	0.120
Solo treatment approaches	4	236	−2.48 (−5.51, 0.55)	0.109	0.099	52.2	
**Control strategy design**							
Placebo control methodology	4	296	−0.85 (−4.13, 2.42)	0.611	0.017	70.5%	0.437
Standard care protocol	2	87	−2.58 (−5.46, 0.30)	0.079	0.346	0.0	
**Okra dosage (mg/day)**							
<4000	2	149	−3.75 (−10.98, 3.49)	0.310	0.024	80.3%	0.363
≥4000	4	234	−0.09 (−3.23, 3.06)	0.958	0.067	58.1%	
**Intervention duration (weeks)**							
≤8	2	147	1.25 (−2.35, 4.84)	0.496	0.144	53.2%	0.120
>8	4	236	−2.48 (−5.51, 0.55)	0.109	0.099	52.2	
**Intervention age (years)**							
<55	3	152	−0.49 (−2.43, 1.45)	0.618	0.881	0.0%	0.563
≥55	3	231	−2.24 (−7.84, 3.36)	0.432	0.002	83.9	
**Country**							
Iran	4	296	−0.85 (−4.13, 2.42)	0.611	0.017	70.5%	0.437
Other countries	2	87	−2.58 (−5.46, 0.30)	0.079	0.346	0.0	
**Baseline BMI**							
Healthy weight or overweight (>30 kg/m^2^)	4	229	−1.23 (−2.86, 0.40)	0.139	0.544	0.0%	0.861
Obese (≥30 kg/m^2^)	2	154	−2.20 (−12.99, 8.57)	0.688	0.002	89.8	
Not reported	0	0	–	–	–	–	
**Sample size**							
≤60	3	113	−2.63 (−8.16, 2.89)	0.350	0.086	59.3%	0.463
>60	3	270	−0.27 (−3.36, 2.82)	0.866	0.031	71.2	
**Publication year**							
≤2022	4	234	−0.09 (−3.23, 3.06)	0.958	0.067	58.1%	0.363
>2022	2	149	−3.75 (−10.98, 3.49)	0.310	0.024	80.3	
**Study quality**							
Poor or fair	3	186	0.28 (−4.52, 5.07)	0.910	0.029	71.9%	0.422
Good	3	197	−2.16 (−5.67, 1.35)	0.228	0.073	61.8	
**Analysis and subgroup results of** okra **supplementation on 2 h PPG**							
**Overall effect**	3	180	−22.39 (−41.39, −3.38)	0.021	<0.001	94.9%	
**Analysis and subgroup results of okra supplementation on fasting insulin**							
**Overall effect**	5	310	0.65 (−0.73, 2.04)	0.356	0.061	55.5%	
**Health condition**							
Individual with diabetes	4	250	0.08 (−1.08, 1.24)	0.892	0.274	22.8%	
Individual with impaired glucose tolerance or prediabetes	1	60	2.40 (0.59, 4.21)	0.009	–	–	
**Intervention approach**							
Standardized okra interventions	3	156	0.54 (−1.05, 2.13)	0.504	0.288	19.7%	0.850
Dietary and lifestyle management approaches	2	154	0.86 (−2.04, 3.76)	0.561	0.011	84.6%	
**Intervention type**							
Capsules and tablets	3	202	0.38 (−1.27, 2.03)	0.652	0.191	39.6%	0.759
Liquid and natural forms	2	108	0.91 (−2.07, 3.89)	0.548	0.027	79.4	
**Treatment pairing p**rotocol							
Pharmaceutical or dietary co‐treatments	2	101	0.49 (−1.60, 2.58)	0.645	0.115	59.8%	0.831
Solo treatment approaches	3	209	0.85 (−1.72, 3.42)	0.516	0.039	69.3	
**Control strategy design**							
Placebo control methodology	4	250	0.08 (−1.08, 1.24)	0.892	0.274	22.8%	0.034
Standard care protocol	1	60	2.40 (0.59, 4.21)	0.009	–	–	
**Okra dosage (mg/day)**							
<4000	3	202	0.38 (−1.27, 2.03)	0.652	0.191	39.6%	0.759
≥4000	2	108	0.91 (−2.07, 3.89)	0.548	0.027	79.4	
**Intervention duration (weeks)**							
≤8	3	152	1.14 (−0.55, 2.83)	0.187	0.081	60.2%	0.131
>8	2	158	−0.54 (−1.91, 0.84)	0.443	0.796	0.0	
**Intervention age (years)**							
<55	4	246	0.65 (−0.83, 2.14)	0.389	0.029	66.6%	0.962
≥55	1	64	0.93 (−10.27, 12.12)	0.871	–	–	
**Country**							
Iran	4	250	0.08 (−1.08, 1.24)	0.892	0.274	22.8%	0.034
Other countries	1	60	2.40 (0.59, 4.21)	0.009	–	–	
**Baseline BMI**							
Healthy weight or overweight (>30 kg/m^2^)	3	186	0.08 (−1.27, 1.44)	0.903	0.145	48.2%	0.133
Obese (≥30 kg/m^2^)	1	64	0.93 (−10.27, 12.12)	0.871	–	–	
Not reported	1	60	2.40 (0.59, 4.21)	0.009	–	–	
**Sample size**							
≤60	3	152	1.14 (−0.55, 2.83)	0.187	0.081	60.2%	0.131
>60	2	158	−0.54 (−1.91, 0.84)	0.443	0.796	0.0	
**Publication year**							
≤2022	1	48	−0.64 (−2.65, 1.37)	0.533	–	–	0.211
>2022	4	262	1.02 (−0.64, 2.68)	0.227	0.062	59.2	
**Study quality**							
Poor or fair	2	113	1.93 (0.68, 3.19)	0.003	0.482	0.0%	0.004
Good	3	197	−0.57 (−1.70, 0.56)	0.324	0.964	0.0	
**Analysis and subgroup results of okra supplementation on FBS**							
**Overall effect**	12	656	−23.66 (−34.20, −13.12)	<0.001	<0.001	95.3%	
**Health condition**							
Individual with diabetes	11	596	−27.84 (−39.84, −15.84)	<0.001	<0.001	94.6%	<0.001
Individual with impaired glucose tolerance or prediabetes	1	60	5.40 (0.37, 10.43)	0.035	–	–	
**Intervention approach**							
Standardized okra interventions	3	156	−6.03 (−39.59, 27.54)	0.725	<0.001	91.5%	0.190
Dietary and lifestyle management approaches	9	500	−29.87 (−41.86, −17.88)	<0.001	<0.001	96.0	
**Intervention type**							
Capsules and tablets	5	378	−15.80 (−34.85, 3.25)	0.104	<0.001	93.3%	0.200
Liquid and natural forms	7	278	−31.87 (−47.37, −16.36)	<0.001	<0.001	96.3	
**Treatment pairing p**rotocol							
Pharmaceutical or dietary co‐treatments	4	260	−13.85 (−40.28, 12.58)	0.304	<0.001	96.3%	0.329
Solo treatment approaches	8	396	−28.43 (−41.07, −15.79)	<0.001	<0.001	94.2	
**Control strategy design**							
Placebo control methodology	5	349	−15.42 (−35.51, 4.67)	0.132	<0.001	93.3%	0.208
Standard care protocol	7	307	−31.55 (−46.64, −16.46)	<0.001	<0.001	96.3	
**Okra dosage (mg/day)**							
<4000	3	202	−13.65 (−52.43, 25.13)	0.490	<0.001	96.6%	0.511
≥4000	9	454	−27.27 (−39.37, −15.18)	<0.001	<0.001	95.2	
**Intervention duration (weeks)**							
≤8	8	420	−21.74 (−35.07, −8.41)	0.001	<0.001	96.4%	0.488
>8	4	236	−28.01 (−39.71, −16.31)	<0.001	0.085	54.7	
**Intervention age (years)**							
<55	9	425	−25.03 (−38.38, −11.69)	<0.001	<0.001	96.4%	0.695
≥55	3	231	−22.01 (−29.17, −14.85)	<0.001	0.340	7.3	
**Country**							
Iran	7	469	−18.89 (−31.99, −5.79)	0.005	<0.001	95.8%	0.124
Other countries	5	187	−46.68 (−79.53, −13.82)	0.005	<0.001	93.4	
**Baseline BMI**							
Healthy weight or overweight (>30 kg/m^2^)	8	382	−26.74 (−42.11, −11.37)	0.001	<0.001	93.1%	0.927
Obese (≥30 kg/m^2^)	2	154	−24.65 (−32.41, −16.89)	<0.001	0.497	0.0	
Not reported	2	120	−17.82 (−63.43, 27.79)	0.444	<0.001	99.2	
**Sample size**							
≤60	9	386	−24.73 (−38.50, −10.95)	<0.001	<0.001	95.9%	0.985
>60	3	270	−24.89 (−35.04, −14.75)	<0.001	0.031	71.2	
**Publication year**							
≤2022	6	274	−41.59 (−62.88, −20.29)	<0.001	<0.001	84.9%	0.038
>2022	6	382	−14.69 (−28.47, −0.90)	0.037	<0.001	97.3	
**Study quality**							
Poor or fair	8	399	−18.71 (−31.24, −6.18)	0.003	<0.001	94.3%	0.089
Good	4	257	−32.40 (−42.01, −22.79)	<0.001	0.029	66.7	
**Analysis and subgroup results of okra supplementation on HbA1c**							
**Overall effect**	9	556	−0.30 (−0.59, −0.02)	0.034	<0.001	77.6%	
**Health condition**							
Individual with diabetes	8	496	−0.34 (−0.65, −0.04)	0.028	<0.001	79.7%	0.284
Individual with impaired glucose tolerance or prediabetes	1	60	0.00 (−0.55, 0.55)	1.000	–	–	
**Intervention approach**							
Standardized okra interventions	3	156	−0.02 (−0.37, 0.33)	0.914	0.162	45.0%	0.096
Dietary and lifestyle management approaches	6	400	−0.45 (−0.81, −0.09)	0.015	<0.001	80.1	
**Intervention type**							
Capsules and tablets	5	378	−0.28 (−0.71, 0.16)	0.218	<0.001	80.2%	0.760
Liquid and natural forms	4	178	−0.38 (−0.88, 0.13)	0.141	0.002	79.8	
**Treatment pairing p**rotocol							
Pharmaceutical or dietary co‐treatments	4	260	−0.20 (−0.51, 0.11)	0.199	0.003	78.9%	0.351
Solo treatment approaches	5	296	−0.54 (−1.18, 0.10)	0.100	<0.001	81.0	
**Control strategy design**							
Placebo control methodology	5	349	−0.32 (−0.71, 0.07)	0.108	0.001	78.1%	0.880
Standard care protocol	4	207	−0.37 (−1.00, 0.25)	0.239	0.001	82.2	
**Okra dosage (mg/day)**							
<4000	3	202	−0.32 (−0.98, 0.34)	0.346	0.003	82.6%	0.967
≥4000	6	354	−0.30 (−0.65, 0.04)	0.085	<0.001	79.2	
**Intervention duration (weeks)**							
≤8	5	320	−0.18 (−0.45, 0.10)	0.206	0.004	73.7%	0.214
>8	4	236	−0.72 (−1.53, 0.09)	0.083	<0.001	83.8	
**Intervention age (years)**							
<55	6	325	−0.33 (−0.72, 0.06)	0.097	<0.001	80.7%	0.917
≥55	3	231	−0.29 (−0.82, 0.23)	0.271	0.025	72.8	
**Country**							
Iran	6	409	−0.31 (−0.57, −0.05)	0.020	0.002	73.2%	0.648
Other countries	3	147	−0.60 (−1.80, 0.60)	0.330	<0.001	87.5	
**Baseline BMI**							
Healthy weight or overweight (>30 kg/m^2^)	5	282	−0.37 (−1.00, 0.25)	0.241	<0.001	85.3%	0.101
Obese (≥30 kg/m^2^)	2	154	−0.57 (−0.81, −0.34)	<0.001	0.622	0.0	
Not reported	2	120	−0.27 (−0.42, −0.12)	<0.001	0.317	0.3	
**Sample size**							
≤60	6	286	−0.25 (−0.61, 0.12)	0.183	0.001	76.2%	0.613
>60	3	270	−0.41 (−0.94, 0.12)	0.127	0.011	77.7	
**Publication year**							
≤2022	4	234	−0.49 (−1.17, 0.19)	0.155	<0.001	86.4%	0.519
>2022	5	322	−0.25 (−0.56, 0.06)	0.119	0.014	68.0	
**Study quality**							
Poor or fair	5	299	−0.33 (−0.93, 0.27)	0.281	<0.001	86.4%	0.956
Good	4	257	−0.35 (−0.62, −0.08)	0.012	0.095	52.9	
**Analysis and subgroup results of okra supplementation on HOMA‐IR**							
**Overall effect**	5	310	−0.59 (−1.01, −0.18)	0.005	0.331	13.0%	
**Health condition**							
Individual with diabetes	4	250	−0.51 (−1.16, 0.13)	0.119	0.228	30.7%	0.654
Individual with impaired glucose tolerance or prediabetes	1	60	−0.70 (−1.21, −0.19)	0.007	–	–	
**Intervention approach**							
Standardized okra interventions	3	156	−0.23 (−0.87, 0.40)	0.468	0.384	0.0%	0.155
Dietary and lifestyle management approaches	2	154	−0.80 (−1.25, −0.35)	0.001	0.416	0.0	
**Intervention type**							
Capsules and tablets	3	202	−0.39 (−1.17, 0.39)	0.330	0.168	43.9%	0.446
Liquid and natural forms	2	108	−0.74 (−1.22, −0.27)	0.002	0.642	0.0	
**Treatment pairing p**rotocol							
Pharmaceutical or dietary co‐treatments	2	101	−0.42 (−1.39, 0.55)	0.393	0.216	34.7%	0.663
Solo treatment approaches	3	209	−0.67 (−1.19, −0.14)	0.012	0.276	22.3	
**Control strategy design**							
Placebo control methodology	4	250	−0.51 (−1.16, 0.13)	0.119	0.228	30.7%	0.654
Standard care protocol	1	60	−0.70 (−1.21, −0.19)	0.007	–	–	
**Okra dosage (mg/day)**							
<4000	3	202	−0.39 (−1.17, 0.39)	0.330	0.168	43.9%	0.446
≥4000	2	108	−0.74 (−1.22, −0.27)	0.002	0.642	0.0	
**Intervention duration (weeks)**							
≤8	3	149	−0.60 (−1.03, −0.16)	0.007	0.361	1.9%	0.984
>8	2	161	−0.58 (−1.78, 0.62)	0.341	0.110	60.8	
**Intervention age (years)**							
<55	4	255	−0.69 (−1.09, −0.28)	0.001	0.377	3.2%	0.222
≥55	1	55	0.06 (−1.07, 1.20)	0.913	–	–	
**Country**							
Iran	4	250	−0.51 (−1.16, 0.13)	0.119	0.228	30.7%	0.654
Other countries	1	60	−0.70 (−1.21, −0.19)	0.007	–	–	
**Baseline BMI**							
Healthy weight or overweight (>30 kg/m^2^)	3	195	−0.69 (−1.45, 0.07)	0.075	0.213	35.3%	0.468
Obese (≥30 kg/m^2^)	1	55	0.06 (−1.07, 1.20)	0.913	−	−	
Not reported	1	60	−0.70 (−1.21, −0.19)	0.007	−	−	
**Sample size**							
≤60	4	216	−0.50 (−0.92, −0.08)	0.019	0.364	5.8%	0.229
>60	1	94	−1.16 (−2.14, −0.18)	0.021	–	–	
**Publication year**							
≤2022	1	48	−1.03 (−2.33, 0.27)	0.119	–	–	0.480
>2022	4	262	−0.53 (−1.02, −0.04)	0.033	0.246	27.7	
**Study quality**							
Poor or fair	2	113	−0.48 (−1.10, 0.15)	0.132	0.212	35.8%	0.633
Good	3	197	−0.72 (−1.49, 0.05)	0.067	0.244	29.2	
**Analysis and subgroup results of** okra **supplementation on hs‐CRP**							
Overall effect	2	149	−1.21 (−3.24, 0.81)	0.239	0.007	86.3%	
**Analysis and subgroup results of** okra **supplementation on HDL‐C**							
**Overall effect**	8	563	3.01 (−0.57, 6.59)	0.099	<0.001	93.0%	
**Health condition**							
Individual with diabetes	6	433	3.12 (−1.48, 7.73)	0.184	<0.001	94.9%	0.954
Individual with impaired glucose tolerance or prediabetes	2	130	3.27 (1.53, 5.01)	<0.001	0.676	0.0	
**Intervention approach**							
Standardized okra interventions	1	48	0.03 (−2.59, 2.65)	0.982	–	–	0.164
Dietary and lifestyle management approaches	7	515	3.48 (−0.61, 7.56)	0.095	<0.001	93.8	
**Intervention type**							
Capsules and tablets	5	395	1.37 (−0.87, 3.61)	0.230	0.001	79.0%	0.504
Liquid and natural forms	3	168	5.68 (−6.77, 18.14)	0.371	<0.001	96.5	
**Treatment pairing p**rotocol							
Pharmaceutical or dietary co‐treatments	3	207	4.30 (−5.53, 14.14)	0.391	<0.001	97.9%	0.688
Solo treatment approaches	5	356	2.27 (0.83, 3.70)	0.002	0.247	26.2	
**Control strategy design**							
Placebo control methodology	5	366	0.72 (−1.31, 2.75)	0.487	0.002	76.2%	0.172
Standard care protocol	3	197	7.57 (−2.04, 17.17)	0.123	<0.001	92.4	
**Okra dosage (mg/day)**							
<4000	3	219	1.94 (0.07, 3.81)	0.042	0.112	54.3%	0.624
≥4000	5	344	3.74 (−3.19, 10.66)	0.290	<0.001	95.8	
**Intervention duration (weeks)**							
≤8	5	337	3.66 (−2.29, 9.61)	0.228	<0.001	95.8%	0.550
>8	3	226	1.75 (−0.22, 3.72)	0.082	0.198	38.3	
**Intervention age (years)**							
<55	5	337	4.61 (−0.79, 10.02)	0.094	<0.001	94.0%	0.191
≥55	3	226	0.47 (−2.58, 3.53)	0.762	0.010	78.3	
**Country**							
Iran	6	426	3.03 (−1.13, 7.18)	0.153	<0.001	94.9%	0.800
Other countries	2	137	3.70 (0.50, 6.91)	0.024	0.631	0.0	
**Baseline BMI**							
Healthy weight or overweight (>30 kg/m^2^)	3	219	1.77 (−0.31, 3.85)	0.095	0.195	38.8%	0.090
Obese (≥30 kg/m^2^)	2	154	−0.95 (−3.15, 1.25)	0.398	0.145	52.9	
Not reported	3	190	7.22 (−2.76, 17.20)	0.156	<0.001	95.7	
**Sample size**							
≤60	4	223	4.32 (−3.99, 12.64)	0.308	<0.001	96.0%	0.555
>60	4	340	1.68 (−1.16, 4.52)	0.246	<0.001	83.5	
**Publication year**							
≤2022	3	224	0.42 (−2.77, 3.61)	0.797	0.011	78.0%	0.178
>2022	5	339	4.66 (−0.63, 9.94)	0.084	<0.001	94.2	
**Study quality**							
Poor or fair	4	223	1.55 (−2.11, 5.21)	0.405	<0.001	83.4%	0.477
Good	4	340	4.27 (−2.26, 10.79)	0.200	<0.001	96.1	
**Analysis and subgroup results of okra supplementation on LDL‐C**							
**Overall effect**	8	563	−8.55 (−14.42, −2.68)	0.004	0.003	68.0%	
**Health condition**							
Individual with diabetes	6	433	−8.85 (−16.12, −1.58)	0.017	0.002	73.9%	0.831
Individual with impaired glucose tolerance or prediabetes	2	130	−7.30 (−19.49, 4.89)	0.241	0.117	59.3	
**Intervention approach**							
Standardized okra interventions	1	48	−5.84 (−19.77, 8.09)	0.411	–	–	0.707
Dietary and lifestyle management approaches	7	515	−8.78 (−15.18, −2.38)	0.007	0.002	72.0	
**Intervention type**							
Capsules and tablets	5	395	−7.29 (−12.41, −2.17)	0.005	0.185	35.4%	0.743
Liquid and natural forms	3	168	−9.72 (−23.41, 3.96)	0.164	0.008	79.2	
**Treatment pairing p**rotocol							
Pharmaceutical or dietary co‐treatments	3	207	−9.35 (−23.33, 4.63)	0.190	<0.001	87.2%	0.881
Solo treatment approaches	5	356	−8.22 (−13.06, −3.38)	0.001	0.328	13.4	
**Control strategy design**							
Placebo control methodology	5	366	−7.78 (−12.99, −2.57)	0.003	0.219	30.4%	0.885
Standard care protocol	3	197	−8.85 (−22.41, 4.72)	0.201	0.002	84.1	
**Okra dosage (mg/day)**							
<4000	3	219	−11.14 (−16.58, −5.70)	<0.001	0.529	0.0%	0.429
≥4000	5	344	−6.69 (−16.27, 2.88)	0.171	<0.001	80.5	
**Intervention duration (weeks)**							
≤8	5	337	−8.60 (−17.52, 0.33)	0.059	0.001	78.7%	0.926
>8	3	226	−8.09 (−13.99, −2.18)	0.007	0.342	6.7	
**Intervention age (years)**							
<55	5	337	−12.05 (−18.81, −5.29)	<0.001	0.042	59.7%	0.042
≥55	3	226	−3.10 (−8.43, 2.22)	0.253	0.714	0.0	
**Country**							
Iran	6	426	−10.37 (−17.07, −3.66)	0.002	0.004	70.6%	0.135
Other countries	2	137	−2.47 (−10.37, 5.44)	0.541	0.645	0.0	
**Baseline BMI**							
Healthy weight or overweight (>30 kg/m^2^)	3	219	−8.33 (−14.64, −2.03)	0.010	0.350	4.7%	0.241
Obese (≥30 kg/m^2^)	2	154	−2.79 (−9.11, 3.52)	0.386	0.424	0.0	
Not reported	3	190	−12.28 (−22.64, −1.93)	0.020	0.017	75.5	
**Sample size**							
≤60	4	223	−9.06 (−19.40, 1.29)	0.086	0.007	75.1%	0.812
>60	4	340	−7.58 (−13.99, −1.16)	0.021	0.105	51.2	
**Publication year**							
≤2022	3	224	−2.65 (−8.32, 3.03)	0.361	0.792	0.0%	0.042
>2022	5	339	−11.75 (−18.46, −5.04)	0.001	0.028	63.3	
**Study quality**							
Poor or fair	4	223	−4.77 (−10.73, 1.19)	0.117	0.204	34.8%	0.102
Good	4	340	−12.73 (−20.18, −5.27)	0.001	0.057	60.1	
**Analysis and subgroup results of** okra **supplementation on TC**							
**Overall effect**	8	563	−12.58 (−22.78, −2.37)	0.016	<0.001	86.8%	
**Health condition**							
Individual with diabetes	6	433	−12.70 (−25.95, 0.55)	0.060	<0.001	90.2%	0.921
Individual with impaired glucose tolerance or prediabetes	2	130	−13.61 (−25.98, −1.24)	0.031	0.201	38.8	
**Intervention approach**							
Standardized okra interventions	2	125	−5.48 (−14.13, 3.17)	0.214	0.634	0.0%	0.232
Dietary and lifestyle management approaches	6	438	−14.66 (−26.99, −2.32)	0.020	<0.001	88.9	
**Intervention type**							
Capsules and tablets	5	395	−10.15 (−19.67, −0.63)	0.037	0.002	76.7%	0.595
Liquid and natural forms	3	168	−16.37 (−37.21, 4.47)	0.124	<0.001	87.3	
**Treatment pairing p**rotocol							
Pharmaceutical or dietary co‐treatments	3	207	−12.90 (−38.38, 12.58)	0.321	<0.001	95.4%	0.998
Solo treatment approaches	5	356	−12.94 (−20.02, −5.86)	<0.001	0.119	45.5	
**Control strategy design**							
Placebo control methodology	5	366	−11.08 (−20.67, −1.49)	0.023	0.003	74.7%	0.787
Standard care protocol	3	197	−14.50 (−37.40, 8.39)	0.214	<0.001	92.0	
**Okra dosage (mg/day)**							
<4000	3	219	−17.50 (−23.42, −11.57)	<0.001	0.438	0.0%	0.391
≥4000	5	344	−9.53 (−26.72, 7.67)	0.277	<0.001	92.2	
**Intervention duration (weeks)**							
≤8	5	337	−12.52 (−27.86, 2.83)	0.110	<0.001	91.2%	0.985
>8	3	226	−12.34 (−23.11, −1.56)	0.025	0.067	62.9	
**Intervention age (years)**							
<55	5	337	−18.74 (−29.08, −8.40)	<0.001	0.001	78.7%	0.019
≥55	3	226	−2.94 (−11.12, 5.24)	0.481	0.181	41.5	
**Country**							
Iran	6	426	−15.08 (−27.00, −3.17)	0.013	<0.001	88.9%	0.149
Other countries	2	137	−3.86 (−13.38, 5.65)	0.426	0.999	0.0	
**Baseline BMI**							
Healthy weight or overweight (>30 kg/m^2^)	3	219	−11.55 (−23.04, −0.06)	0.049	0.059	64.7%	0.264
Obese (≥30 kg/m^2^)	2	154	−3.11 (−17.28, 11.06)	0.667	0.069	69.8	
Not reported	3	190	−20.31 (−35.35, −5.27)	0.008	0.001	85.0	
**Sample size**							
≤60	4	223	−15.24 (−31.32, 0.84)	0.063	<0.001	85.5%	0.603
>60	4	340	−9.96 (−21.69, 1.77)	0.096	0.001	82.5	
**Publication year**							
≤2022	3	224	−1.41 (−8.01, 5.19)	0.675	0.341	7.0%	0.003
>2022	5	339	−19.23 (−29.16, −9.30)	<0.001	0.001	77.9	
**Study quality**							
Poor or fair	4	223	−5.80 (−16.59, 4.98)	0.292	0.011	73.2%	0.102
Good	4	340	−19.53 (−31.94, −7.12)	0.002	0.001	82.0	
**Analysis and subgroup results of** okra **supplementation on TG**							
**Overall effect**	9	616	−10.74 (−26.69, 5.22)	0.187	<0.001	89.8%	
**Health condition**							
Individual with diabetes	7	486	−11.71 (−30.58, 7.16)	0.224	<0.001	91.5%	0.827
Individual with impaired glucose tolerance or prediabetes	2	130	−9.05 (−23.62, 5.52)	0.223	0.518	0.0	
**Intervention approach**							
Standardized okra interventions	3	178	−5.58 (−23.53, 12.38)	0.543	0.233	31.4%	0.546
Dietary and lifestyle management approaches	6	438	−13.78 (−33.43, 5.87)	0.169	<0.001	92.5	
**Intervention type**							
Capsules and tablets	6	448	−5.61 (−18.33, 7.11)	0.388	0.018	63.5%	0.185
Liquid and natural forms	3	168	−22.87 (−44.96, −0.77)	0.043	0.012	77.3	
**Treatment pairing paradigm**							
Pharmaceutical or dietary co‐treatments	4	260	−8.86 (−40.37, 22.65)	0.582	<0.001	95.1%	0.784
Solo treatment approaches	5	356	−13.42 (−21.85, −4.99)	0.002	0.673	0.0	
**Control strategy design**							
Placebo control methodology	6	419	−7.96 (−20.92, 5.01)	0.229	0.011	66.5%	0.607
Standard care protocol	3	197	−16.43 (−45.97, 13.12)	0.276	0.001	84.7	
**Okra dosage (mg/day)**							
<4000	4	272	−13.41 (−23.99, −2.83)	0.013	0.295	19.1%	0.836
≥4000	5	344	−10.41 (−36.68, 15.86)	0.437	<0.001	93.6	
**Intervention duration (weeks)**							
≤8	6	390	−8.32 (−31.32, 14.69)	0.479	<0.001	92.9%	0.571
>8	3	226	−15.61 (−25.95, −5.28)	0.003	0.495	0.0	
**Intervention age (years)**							
<55	6	390	−15.27 (−31.32, 0.77)	0.062	<0.001	84.5%	0.358
≥55	3	226	−2.94 (−23.80, 17.92)	0.782	0.043	68.2	
**Country**							
Iran	7	479	−12.90 (−30.88, 5.08)	0.160	<0.001	91.5%	0.412
Other countries	2	137	−1.87 (−21.18, 17.45)	0.850	0.881	0.0	
**Baseline BMI**							
Healthy weight or overweight (>30 kg/m^2^)	4	272	−11.17 (−23.04, 0.70)	0.065	0.269	23.6%	0.742
Obese (≥30 kg/m^2^)	2	154	−4.22 (−38.04, 29.60)	0.807	0.014	83.5	
Not reported	3	190	−19.62 (−44.03, 4.79)	0.115	0.001	86.4	
**Sample size**							
≤60	5	276	−16.72 (−36.24, 2.80)	0.093	0.001	78.7%	0.347
>60	4	340	−5.01 (−19.67, 9.65)	0.503	0.017	70.5	
**Publication year**							
≤2022	3	224	−1.87 (−20.82, 17.09)	0.847	0.054	65.8%	0.267
>2022	6	392	−15.96 (−32.07, 0.16)	0.052	<0.001	83.9	
**Study quality**							
Poor or fair	5	276	1.93 (−9.02, 12.89)	0.730	0.245	26.6%	0.003
Good	4	340	−25.74 (−40.07, −11.41)	<0.001	0.004	77.2	
**Analysis and subgroup results of** okra **supplementation on ALP**							
**Overall effect**	2	164	2.73 (−2.41, 7.87)	0.298	0.290	10.5%	
**Analysis and subgroup results of** okra **supplementation on ALT**							
**Overall effect**	6	453	−1.37 (−4.42, 1.68)	0.380	<0.001	81.4%	
**Health condition**							
Individual with diabetes	4	323	0.14 (−1.26, 1.54)	0.844	0.658	0.0%	0.158
Individual with impaired glucose tolerance or prediabetes	2	130	−5.11 (−12.26, 2.04)	0.161	0.003	88.6	
**Intervention approach**							
Standardized okra interventions	1	53	1.79 (−1.49, 5.07)	0.285	–	–	0.118
Dietary and lifestyle management approaches	5	400	−2.02 (−5.49, 1.45)	0.253	<0.001	82.5	
**Intervention type**							
Capsules and tablets	5	393	−1.34 (−5.00, 2.33)	0.476	<0.001	85.1%	0.980
Liquid and natural forms	1	60	−1.40 (−5.05, 2.25)	0.452	–	–
**Treatment pairing p**rotocol							
Pharmaceutical or dietary co‐treatments	2	152	0.23 (−1.86, 2.32)	0.828	0.235	29.1%	0.328
Solo treatment approaches	4	301	−2.40 (−7.22, 2.43)	0.331	<0.001	84.4	
**Control strategy design**							
Placebo control methodology	4	316	−1.79 (−5.87, 2.28)	0.388	<0.001	88.5%	0.689
Standard care protocol	2	137	−0.73 (−3.96, 2.49)	0.657	0.442	0.0	
**Okra dosage (mg/day)**							
<4000	3	217	−2.27 (−8.60, 4.07)	0.483	<0.001	91.8%	0.608
≥4000	3	236	−0.56 (−2.18, 1.06)	0.499	0.739	0.0	
**Intervention duration (weeks)**							
≤8	4	277	2.18 (−6.35, 1.99)	0.305	<0.001	88.0%	0.318
>8	2	176	0.36 (−2.40, 3.13)	0.795	0.688	0.0	
**Intervention age (years)**							
<55	4	277	−2.06 (−6.72, 2.60)	0.387	<0.001	87.7%	0.504
≥55	2	176	−0.35 (−2.16, 1.46)	0.703	0.554	0.0	
**Country**							
Iran	4	316	−1.79 (−5.87, 2.28)	0.388	<0.001	88.5%	0.689
Other countries	2	137	−0.73 (−3.96, 2.50)	0.657	0.442	0.0	
**Baseline BMI**							
Healthy weight or overweight (>30 kg/m^2^)	3	224	0.96 (−1.16, 3.07)	0.375	0.747	0.0%	0.221
Obese (≥30 kg/m^2^)	1	99	−0.50 (−2.37, 1.37)	0.601	−	–	
Not reported	2	130	−5.11 (−12.26, 2.04)	0.161	0.003	88.6	
**Sample size**							
≤60	2	113	0.30 (−2.82, 3.42)	0.851	0.203	38.3%	0.376
>60	4	340	−2.13 (−6.53, 2.26)	0.341	<0.001	86.8	
**Publication year**							
≤2022	2	176	−0.35 (−2.16, 1.46)	0.703	0.554	0.0%	0.504
>2022	4	277	−2.06 (−6.72, 2.60)	0.387	<0.001	87.7	
**Study quality**							
Poor or fair	5	359	−1.66 (−5.41, 2.09)	0.386	<0.001	84.5%	0.469
Good	1	94	0.12 (−2.90, 3.14)	0.938	–	–
**Analysis and subgroup results of okra supplementation on AST**							
**Overall effect**	5	393	−1.51 (−3.61, 0.59)	0.159	0.002	76.1%	
**Health condition**							
Individual with diabetes	4	323	−0.52 (−1.95, 0.90)	0.472	0.160	41.9%	0.001
Individual with impaired glucose tolerance or prediabetes	1	70	−5.68 (−8.49, −2.87)	<0.001	–	–	
**Intervention approach**							
Standardized okra interventions	1	53	0.93 (−0.83, 2.69)	0.300	–	–	0.028
Dietary and lifestyle management approaches	4	340	−2.22 (−4.41, −0.03)	0.047	0.026	67.7	
**Intervention type**							
Capsules and tablets	5	393	−1.51 (−3.61, 0.59)	0.159	0.002	76.1%	
Liquid and natural forms	0	0	–	–	–	–	
**Treatment pairing paradigm**							
Pharmaceutical or dietary co‐treatments	2	152	−0.52 (−3.39, 2.35)	0.723	0.025	80.2%	0.427
Solo treatment approaches	3	241	−2.35 (−5.84, 1.14)	0.187	0.010	78.4	
**Control strategy design**							
Placebo control methodology	4	316	−1.87 (−4.61, 0.87)	0.181	0.001	81.3%	0.383
Standard care protocol	1	77	−0.33 (−2.42, 1.76)	0.755	–	–	
**Okra dosage (mg/day)**							
<4000	3	217	−1.88 (−6.16, 2.40)	0.389	<0.001	86.9%	0.784
≥4000	2	176	−1.24 (−2.87, 0.39)	0.136	0.243	26.8	
**Intervention duration (weeks)**							
≤8	3	217	−2.10 (−5.52, 1.32)	0.228	<0.001	87.5%	0.422
>8	2	176	−0.51 (−2.33, 1.31)	0.579	0.730	0.0	
**Intervention age (years)**							
<55	3	217	−1.88 (−6.16, 2.40)	0.389	<0.001	86.9%	0.784
≥55	2	176	−1.24(−2.87, 0.39)	0.136	0.243	26.8	
**Country**							
Iran	4	316	−1.87 (−4.61, 0.87)	0.181	0.001	81.3%	0.383
Other countries	1	77	−0.33 (−2.42, 1.76)	0.755	–	–	
**Baseline BMI**							
Healthy weight or overweight (>30 kg/m^2^)	3	224	0.23 (−1.03, 1.50)	0.719	0.504	0.0%	<0.001
Obese (≥30 kg/m^2^)	1	99	−2.00 (−3.86, −0.14)	0.035	−	–	
Not reported	1	70	−5.68 (−8.49, −2.87)	<0.001	−	–	
**Sample size**							
≤60	1	53	0.93 (−0.83, 2.69)	0.300	–	–	0.028
>60	4	340	−2.22 (−4.41, −0.03)	0.047	0.026	67.7	
**Publication year**							
≤2022	2	176	−1.24 (−2.87, 0.39)	0.136	0.243	26.8%	0.784
>2022	3	213	−1.88 (−6.16, 2.40)	0.389	<0.001	86.9	
**Study quality**							
Poor or fair	4	299	−1.61 (−4.06, 0.85)	0.199	0.001	82.1%	0.815
Good	1	94	−1.08 (−4.77, 2.61)	0.567	–	–	
**Analysis and subgroup results of** okra **supplementation on BUN**							
**Overall effect**	4	294	0.24 (−0.39, 0.87)	0.458	0.365	5.7%	
**Analysis and subgroup results of** okra supplementation on creatinine							
**Overall effect**	5	354	−0.01 (−0.04, 0.01)	0.169	0.471	0.0%	
**Health condition**							
Individual with diabetes	3	224	−0.02 (−0.05, 0.02)	0.320	0.225	33.0%	0.606
Individual with impaired glucose tolerance or prediabetes	**2**	**130**	**−0.01 (−0.04, 0.04)**	**0.879**	**0.817**	**0.0%**	
**Intervention approach**							
Standardized okra interventions	2	130	0.00 (−0.03, 0.04)	0.833	0.803	0.0%	0.229
Dietary and lifestyle management approaches	3	224	−0.02 (−0.05, 0.00)	0.072	0.364	1.0	
**Intervention type**							
Capsules and tablets	4	294	−0.01 (−0.04, 0.01)	0.263	0.317	14.9%	0.919
Liquid and natural forms	1	60	−0.01 (−0.08, 0.06)	0.782	–	–	
**Treatment pairing p**rotocol							
Pharmaceutical or dietary co‐treatments	1	53	0.01 (−0.05, 0.07)	0.744	–	–	0.387
Solo treatment approaches	4	301	−0.02 (−0.04, 0.00)	0.111	0.424	0.0	
**Control strategy design**							
Placebo control methodology	3	217	−0.02 (−0.05, 0.02)	0.317	0.225	33.0%	0.603
Standard care protocol	2	137	−0.01 (−0.04, 0.04)	0.886	0.812	0.0	
**Okra dosage (mg/day)**							
<4000	3	217	−0.02 (−0.05, 0.02)	0.317	0.225	33.0%	0.603
≥4000	2	137	−0.01 (−0.04, 0.04)	0.886	0.812	0.0	
**Intervention duration (weeks)**							
≤8	3	217	0.00 (−0.03, 0.03)	0.961	0.913	0.0%	0.353
>8	2	137	−0.02 (−0.06, 0.02)	0.242	0.175	45.7	
**Intervention age (years)**							
<55	4	277	−0.02 (−0.04, 0.00)	0.131	0.384	1.7%	0.488
≥55	1	77	0.00 (−0.05, 0.05)	0.990	–	–	
**Country**							
Iran	3	217	−0.02 (−0.05, 0.02)	0.317	0.225	33.0%	0.603
Other countries	2	137	−0.01 (−0.04, 0.04)	0.886	0.812	0.0	
**Baseline BMI**							
Healthy weight or overweight (>30 kg/m^2^)	3	224	−0.02 (−0.05, 0.02)	0.320	0.225	33.0%	0.606
Obese (≥30 kg/m^2^)	0	0	–	–	−	–	
Not reported	2	130	−0.01 (−0.04, 0.04)	0.879	0.817	0.0	
**Sample size**							
≤60	2	113	0.00 (−0.04, 0.05)	0.945	0.673	0.0%	0.488
>60	3	241	−0.02 (−0.05, 0.01)	0.227	0.254	26.9	
Publication year							
≤2022	1	77	0.00 (−0.05, 0.05)	0.990	–	–	0.488
>2022	4	277	−0.02 (−0.04, 0.00)	0.131	0.384	1.7	
**Study quality**							
Poor or fair	4	260	0.00 (−0.03, 0.03)	0.962	0.981	0.0%	0.067
Good	1	94	−0.04 (−0.07, −0.01)	0.022	–	–	

Abbreviations: 2 h PPG, 2 h postprandial glucose; BMI, body mass index; DBP, diastolic blood pressure; FBS, fasting blood sugar; HbA1c, haemoglobin A1C; HC, hip circumference; HDL‐C, high‐density lipoprotein cholesterol; HOMA‐IR, homeostatic model assessment of insulin resistance; hs‐CRP, high‐sensitivity C‐reactive protein; LDL‐C, low‐density lipoprotein cholesterol; MDA, malondialdehyde; QUICKI, quantitative insulin sensitivity check index; SBP, systolic blood pressure; TC, total cholesterol; TG, triglycerides; WC, waist circumference; WHR, waist‐to‐hip ratio; WMD, weighted mean difference.

Considering the intervention approach, HbA1c, HOMA‐IR, LDL‐C, TC and AST were significantly improved when okra was administered as part of dietary and lifestyle management strategies. FBS, however, exhibited significant effects regardless of the approach.

Regarding intervention type, liquid and natural forms of okra significantly improved FBS, HOMA‐IR and TG, whereas capsule and tablet formulations were more effective in lowering LDL‐C and TC.

In subgroup analysis by treatment pairing protocol, solo treatment approaches resulted in significant reductions in FBS, HOMA‐IR, HDL‐C, LDL‐C, TC and TG.

In terms of control strategy design, insulin, FBS and HOMA‐IR were significantly altered when compared with standard care protocols. Conversely, LDL‐C and TC showed significant improvements in studies using placebo‐controlled methodologies.

Analysis by dosage indicated that lower doses (<4000 mg/day) were associated with improvements in HDL‐C, LDL‐C, TC and TG, whereas higher doses (≥4000 mg/day) significantly affected FBS and HOMA‐IR.

When considering intervention duration, longer interventions (<8 weeks) led to significant changes in LDL‐C, TC and TG, whereas shorter interventions (≤8 weeks) predominantly influenced HOMA‐IR. FBS demonstrated significant alterations in both subgroups.

Subgrouping by participant age revealed that HOMA‐IR, LDL‐C and TC were significantly altered in individuals <55 years of age, while FBS was significantly improved in both younger and older participants.

When stratified by country, HbA1c, LDL‐C and TC showed significant improvements in studies conducted in Iran, whereas insulin, HOMA‐IR and HDL‐C improved in trials from other countries. FBS was significantly affected across both categories.

Based on baseline BMI, LDL‐C and TC were significantly improved in participants with a healthy or overweight status, while HbA1c and AST were significantly altered in obese participants. FBS remained significant in both BMI subgroups.

Examining sample size, trials with larger sample sizes reported significant improvements in HOMA‐IR, LDL‐C and AST, while FBS changes were consistent across both larger and smaller studies.

In terms of publication year, HbA1c, HOMA‐IR, LDL‐C and TC were significantly affected in studies published from 2022 onwards. FBS showed significant changes irrespective of publication year.

Finally, according to study quality, HbA1c, LDL‐C, TC, TG and creatinine were significantly changed in studies with good quality. Insulin was significantly altered only in poor‐ or fair‐quality trials, whereas FBS exhibited significant effects in both categories.

### 3.12 Meta‐regression and non‐linear dose–response analysis

A non‐linear dose–response model was used to investigate the relationship between okra dosage or duration and outcomes. The dose–response analysis showed a significant relationship between okra dose and both FBS (*P* = 0.017) and HbA1c (*P* = 0.005), with an optimal dose of 50 000 mg/day, suggesting that higher doses might enhance reductions in these parameters (Figures S9 and S10). The meta‐regression analysis identified a significant association between okra dosage and both BMI (*P* = 0.020) and HDL‐C (*P *= 0.003; Figures S11 and S12).

### 3.13 GRADE assessment

The GRADE evaluation of okra supplementation effects on metabolic parameters is presented in Table 4. The majority of outcomes, such as BMI, WC, weight, SBP, 2 h PPG, fasting insulin, FBS, HOMA‐IR, hs‐CRP, HDL‐C, LDL‐C, TC, TG, ALP, ALT, AST, BUN and creatinine, were assigned very low certainty of evidence. In contrast, evidence for DBP and HbA1c was considered of low quality.

**TABLE 4:** GRADE profile of okra supplementation on metabolic risk factors in diabetes.

Outcome	Risk of bias	Inconsistency	Indirectness	Imprecision	Publication bias	Number (INT/CON)	WMD (95% CI)	Quality of evidence
BMI	Not serious	Not serious	Very serious^a^	Very serious^[Link]^, ^[Link]^	Publication bias strongly suspected^d^	185/174	−0.21 (−0.70, 0.27)	⨁◯◯◯ Very low
WC	Serious^e^	Not serious	Very serious^a^	Extremely serious^[Link]^, ^[Link]^	None	80/74	0.40 (−1.56, 2.35)	⨁◯◯◯ Very low
Weight	Not serious	Not serious	Very serious^a^	Extremely serious^[Link]^, ^[Link]^	None	85/73	−0.12 (−2.44, 2.19)	⨁◯◯◯ Very low
DBP	Not serious	Not serious	Serious^a^	Serious^[Link]^, ^[Link]^	None	196/187	−0.70 (−1.86, 0.46)	⨁⨁◯◯ Low
SBP	Not serious	Serious^f^	Serious^a^	Serious^[Link]^, ^[Link]^	None	196/187	−1.08 (−3.61, 1.45)	⨁◯◯◯ Very low
2 h PPG	Serious^e^	Very serious^f^	Serious^a^	Serious^c^	None	90/90	−22.39 (−41.39, −3.38)	⨁◯◯◯ Very low
Fasting insulin	Not serious	Serious^f^	Very serious^a^	Very serious^[Link]^, ^[Link]^	None	160/150	0.65 (−0.73, 2.04)	⨁◯◯◯ Very low
FBS	Serious^e^	Very serious^f^	Serious^a^	Not serious	Publication bias strongly suspected^d^	337/319	−23.66 (−34.20, −13.12)	⨁◯◯◯ Very low
HbA1c	Not serious	Serious^f^	Serious^a^	Not serious	None	283/273	−0.30 (−0.59, −0.02)	⨁⨁◯◯ Low
HOMA‐IR	Not serious	Not serious	Very serious^a^	Serious^c^	None	160/150	−0.59 (−1.01, −0.18)	⨁◯◯◯ Very low
hs‐CRP	Not serious	Very serious^f^	Very serious^a^	Very serious^[Link]^, ^[Link]^	None	78/71	−1.21 (−3.24, 0.81)	⨁◯◯◯ Very low
HDL‐C	Not serious	Very serious^f^	Very serious^a^	Serious^b^	None	286/277	3.01 (−0.57, 6.59)	⨁◯◯◯ Very low
LDL‐C	Not serious	Serious^f^	Very serious^a^	Not serious	Publication bias strongly suspected^d^	286/277	−8.55 (−14.42, −2.68)	⨁◯◯◯ Very low
TC	Not serious	Very serious^f^	Very serious^a^	Not serious	Publication bias strongly suspected^d^	288/277	−12.58 (−22.78, −2.37)	⨁◯◯◯ Very low
TG	Not serious	Very serious^f^	Very serious^a^	Serious^b^	Publication bias strongly suspected^d^	313/303	−10.74 (−26.69, 5.22)	⨁◯◯◯ Very low
ALP	Serious^e^	Not serious	Very serious^a^	Very serious^[Link]^, ^[Link]^	None	83/81	2.73 (−2.41, 7.87)	⨁◯◯◯ Very low
ALT	Serious^e^	Very serious^f^	Very serious^a^	Serious^b^	None	228/225	−1.37 (−4.42, 1.68)	⨁◯◯◯ Very low
AST	Not serious	Serious^f^	Very serious^a^	Very serious^[Link]^, ^[Link]^	None	198/195	−1.51 (−3.61, 0.59)	⨁◯◯◯ Very low
BUN	Not serious	Not serious	Very serious^a^	Very serious^[Link]^, ^[Link]^	None	148/146	0.24 (−0.39, 0.87)	⨁◯◯◯ Very low
Creatinine	Not serious	Not serious	Very serious^a^	Very serious^[Link]^, ^[Link]^	None	178/176	−0.01 (−0.04, 0.01)	⨁◯◯◯ Very low

Abbreviations: 2 h PPG, 2 h postprandial glucose; ALP, alkaline phosphatase; ALT, alanine aminotransferase; AST, aspartate aminotransferase; BMI, body mass index; BUN, blood urea nitrogen; CI, confidence interval; DBP, diastolic blood pressure; FBS, fasting blood sugar; HbA1c, haemoglobin A1c; HDL‐C, high‐density lipoprotein cholesterol; HOMA‐IR, homeostatic model assessment of insulin resistance; hs‐CRP, high‐sensitivity C‐reactive protein; INT, intervention group; LDL‐C, low‐density lipoprotein cholesterol; SBP, systolic blood pressure; TC, total cholesterol; TG, triglycerides; WC, waist circumference; WHR, waist‐to‐hip ratio; WMD, weighted mean difference.

^a^Downgraded for indirectness in the country.

^b^Downgraded because the 95% CI crosses the threshold of interest.

^c^Downgraded because the number of participants included was <400.

^d^Publication bias was detected through Egger and Begg's test (*P* < 0.05).

^e^Downgraded because >50% of the participants were from studies with a high risk of bias.

^f^The *I* ^2^ value was >50% (or heterogeneity among the studies was high).

## 4 DISCUSSION

### 4.1 Summary of findings

**FIGURE 2:**
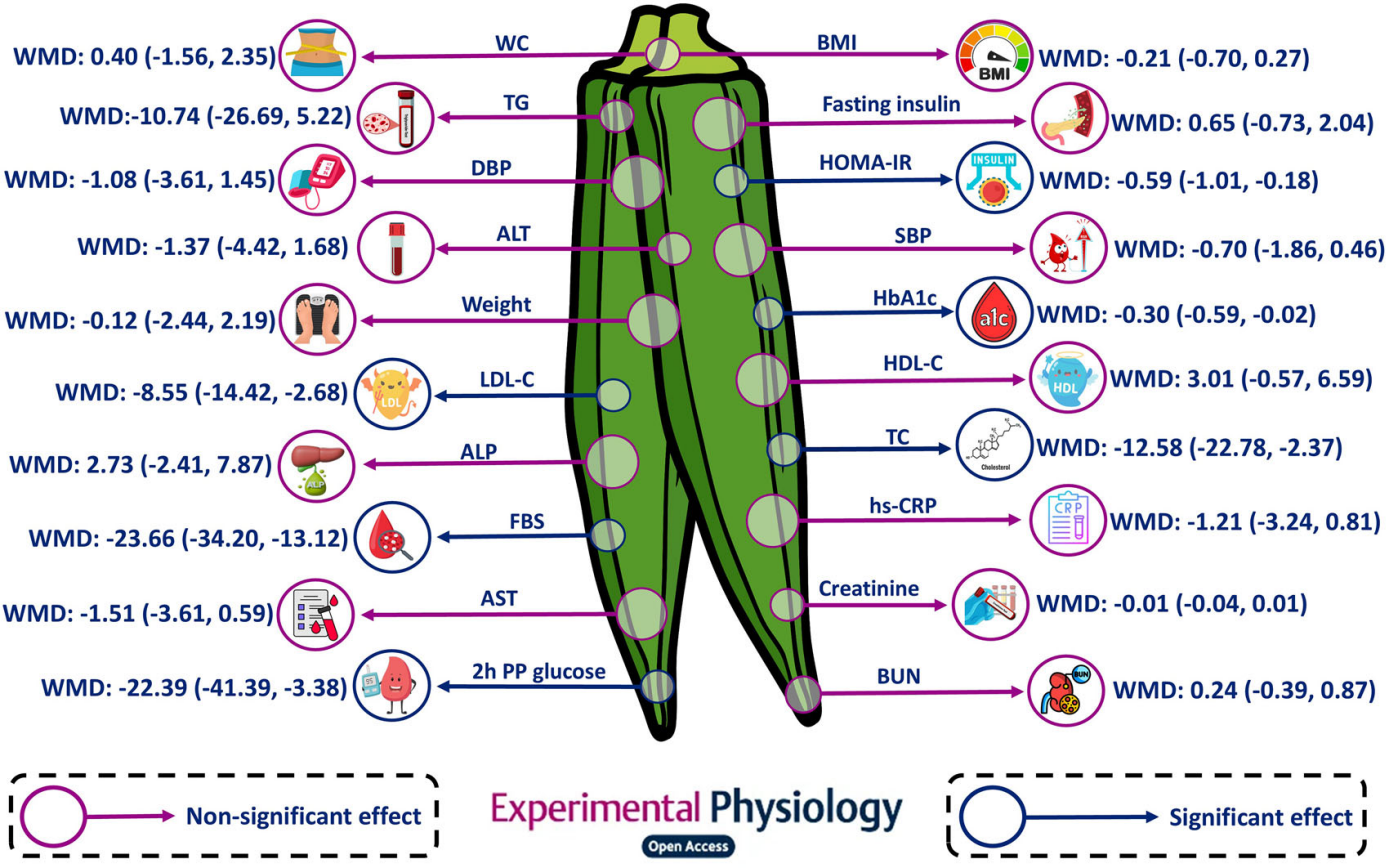
Effects of okra on metabolic risk factors. Abbreviations: 2 h PP glucose, 2 h postprandial glucose; ALP, alkaline phosphatase; ALT, alanine aminotransferase; AST, aspartate aminotransferase; BMI, body mass index; BUN, blood urea nitrogen; DBP, diastolic blood pressure; FBS, fasting blood sugar; HbA1c, haemoglobin A1c; HDL‐C, high‐density lipoprotein cholesterol; HOMA‐IR, homeostatic model assessment for insulin resistance; hs‐CRP, high‐sensitivity C‐reactive protein; LDL‐C, low‐density lipoprotein cholesterol; SBP, systolic blood pressure; TC, total cholesterol; TG, triglycerides; WC, waist circumference; WMD, weighted mean difference.

Our systematic review and meta‐analysis of 14 RCTs published between 2020 and 2025, involving 836 participants across four countries, found that okra supplementation did not significantly affect anthropometric indices (BMI, weight and WC) or blood pressure (SBP and DBP). Subgroup analyses suggested that shorter interventions (<8 weeks) modestly improved HOMA‐IR, whereas longer interventions (>8 weeks) produced clearer benefits in lipid outcomes. Consistent glycaemic improvements were observed, including reductions in FBS, 2 h PPG, HbA1c and HOMA‐IR, although fasting insulin remained unchanged. Lipid outcomes indicated decreases in LDL‐C and TC, whereas HDL‐C and TG did not improve significantly, with stronger effects seen with lower doses (<4000 mg/day), natural formulations and solo treatment. Inflammatory markers (hs‐CRP) and hepatic/renal indices (ALT, AST, ALP, BUN and creatinine) were largely unaffected, with only isolated sensitivity analyses suggesting benefit. Meta‐regression showed dose‐dependent associations with BMI, HDL, FBS and HbA1c, with optimal glycaemic effects at ∼50 000 mg/day. Subgroup analyses further indicated that individuals with diabetes, obese participants, younger adults (<55 years) and studies conducted in Iran demonstrated greater improvements, particularly in FBS, HbA1c, LDL‐C and TC. Nonetheless, GRADE assessments rated most outcomes as very low certainty, with only DBP and HbA1c reaching low‐quality evidence (Figure 2).

### 4.2 Comparison with previous studies

Several prior investigations corroborate the results of our meta‐analysis. Mokgalaboni et al. (2023) synthesized eight RCTs conducted in individuals with T2DM and prediabetes, reporting significant reductions in FBS. These findings align with our results, which also demonstrated improvements in glycaemic control (particularly in FBS), although our pooled analyses revealed modest effects on HbA1c. Likewise, Bahari et al. (2024) reviewed nine RCTs and identified reductions in TC, LDL‐C, FBS and HbA1c, while observing no significant changes in TG, HDL‐C, HOMA‐IR, SBP, DBP or anthropometric outcomes. Their conclusions mirror our observations on glycaemic and lipid parameters and support the efficacy of lower doses (≤3000 mg/day) in driving more pronounced improvements. In preclinical research, Sereno et al. (2022) synthesized evidence from 11 animal studies and demonstrated that okra supplementation improved glycaemic indices, including HbA1c, HOMA‐IR, glucose tolerance and blood glucose, alongside cholesterol reduction. Likewise, Nikpayam et al. (2021) summarized 54 human and animal studies, confirming favourable effects on both glycaemic control and lipid metabolism. At the clinical trial level, one study in prediabetic individuals reported significant reductions in TC, LDL‐C, ALT and uric acid, together with an increase in HDL‐C after 8 weeks of okra supplementation at 3000 mg/day (Afsharmanesh et al., 2024).

In our previous study, we included 12 RCTs and reported significant reductions in BMI, body weight, FM, HC, fasting insulin, FBS, HbA1c, HOMA‐IR, LDL‐C, TC and AST. However, we found no significant effects on SBP, DBP, HDL‐C, TG, ALT, ALP or creatinine (Jafari et al., 2025). Unlike the present analysis, which focused specifically on okra supplementation in patients with diabetes, the earlier study applied broader eligibility criteria that encompassed heterogeneous populations. Differences in sample size and total patient enrolment are likely to have influenced statistical power and pooled effect estimates, and the disproportionate impact of individual trials with larger effect sizes might also have shaped the outcomes. These methodological differences might account for why that synthesis identified consistent reductions in anthropometric measures, whereas ours did not.

At the trial level, Saatchi et al. (2022) reported significant reductions in HbA1c, FBS and blood glucose after 8 weeks of 4000 mg/day okra supplementation in T2DM patients, but no significant changes in HDL‐C, LDL‐C, TC, TG, BMI, WC, AST, ALT, SBP or DBP. This partial overlap with our findings suggests that variations in the study population and relatively short intervention duration might have constrained the detection of broader metabolic effects. Likewise, Nikpayam et al. (2022) evaluated 125 mg/day of dried okra in patients with diabetic nephropathy over 10 weeks and observed reduced energy and carbohydrate intake, without effects on body composition or anthropometry. The advanced renal status of this population is likely to have limited the potential for measurable improvements in anthropometric or lipid outcomes, diverging from our broader results. Peng et al. (2022) investigated a composite formulation (IQP‐AE‐103, combining dehydrated okra powder and inulin) and reported modest weight loss accompanied by shifts in gut microbiota. The addition of inulin (a prebiotic fibre with established metabolic effects) might explain the differences in anthropometric outcomes compared with trials evaluating okra alone. Preclinical evidence also illustrates divergence. Fan et al. (2014) found that okra improved glucose tolerance, reduced TG and altered liver morphology in obese mice, whereas Anjani et al. (2018) reported that neither green nor purple okra extracts significantly affected body weight in diabetic rats, despite both extracts repairing streptozotocin‐induced β‐cell damage, with purple okra demonstrating greater potency. Such discrepancies are likely to stem from strain‐specific differences in animal models, variability in extract composition and heterogeneity in dosing regimens.

### 4.3 Mechanisms of action

Okra modulates glucose regulation through multiple complementary mechanisms. Its high content of soluble fibre and mucilage slows gastric emptying and intestinal glucose absorption, while inhibition of α‐amylase and α‐glucosidase reduces postprandial carbohydrate digestion and uptake (Abbas et al., 2018; Amadi et al., 2021; Fan et al., 2014; Tavakolizadeh et al., 2023). Bioactive flavonoids, such as ursolic acid and quercetin, further enhance insulin sensitivity and glucose utilization by modulating insulin secretion and improving pancreatic β‐cell function through antioxidant activity, thereby mitigating reactive oxygen species (Patel & Barnes, 2010; Sabitha et al., 2012; Xia et al., 2015). Okra also activates peroxisome proliferator‐activated receptor (PPAR)‐dependent pathways, contributing to β‐cell repair and the regulation of glucose and lipid metabolism in both the pancreas and liver (Erfani Majd et al., 2018). Additionally, one of its key polysaccharides, *Abelmoschus esculentus* polysaccharide‐P‐1 (AeP‐P‐1), stimulates the phosphoinositide 3‐kinase (PI3K)/protein kinase B (Akt) signalling pathway in hepatic tissue, promoting glycogen storage and partly restoring renal and hepatic function in diabetic models (Geng et al., 2022).

With respect to lipid metabolism, the soluble fibre in okra binds bile acids and cholesterol in the intestine, promoting their excretion and lowering circulating lipid levels (Nikpayam et al., 2022; Panighel et al., 2022). It also regulates transcription factors, such as sterol regulatory element‐binding protein 1c (SREBP1c) and fatty acid synthase, thereby reducing lipogenesis, while modulation of cholesterol 7 alpha‐hydroxylase and the PPAR–Niemann‐Pick C1‐Like 1 pathway enhances cholesterol catabolism and limits intestinal absorption (Esmaeilzadeh et al., 2020; Fan et al., 2024; Wang et al., 2014). A reduction in circulating non‐esterified fatty acids further decreases lipogenic drive and improves insulin resistance (Fan et al., 2024; Mokgalaboni et al., 2024). Antioxidant constituents (including quercetin, polyphenols and vitamins A and C) prevent lipid oxidation by protecting LDL‐C from oxidative modification, while saponins inhibit cholesterol absorption. These combined actions contribute to decreases in TC and LDL‐C, with possible increases in HDL‐C (Mohammed et al., 2017; Panighel et al., 2022; Patel & Barnes, 2010; Xia et al., 2015; Yang et al., 2022). Enhanced clearance of LDL‐C particles through upregulation of LDL‐C receptor activity provides an additional mechanism (Panighel et al., 2022). Evidence also suggests that lower doses of okra (≤3000 mg/day) might yield more favourable lipid outcomes, potentially owing to improved absorption and reduced gastrointestinal side effects (Tavakolizadeh et al., 2023).

**FIGURE 3:**
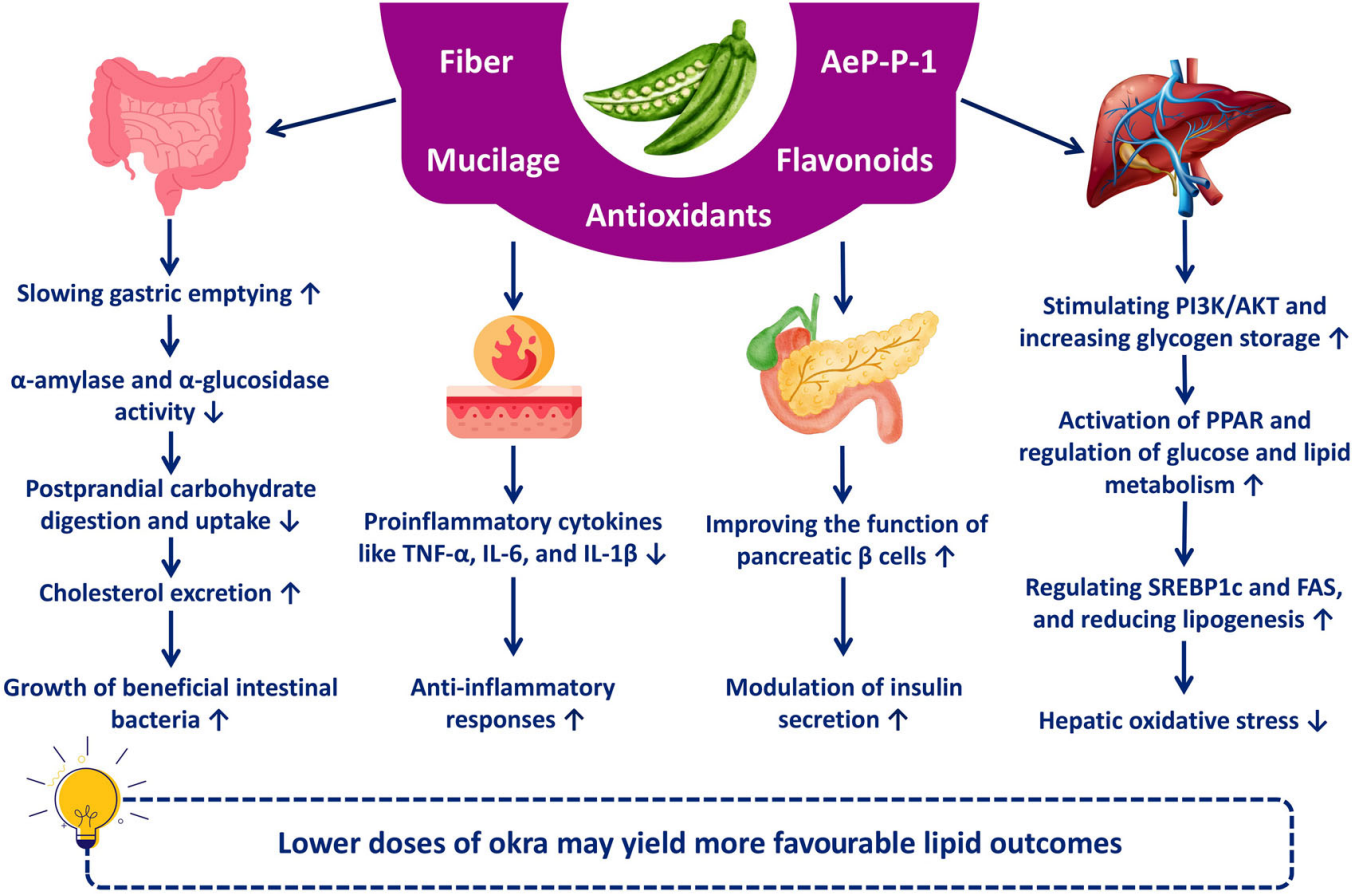
Mechanisms of how okra affects metabolic risk factors. Abbreviations: IL‐6, interleukin‐6; IL‐1β, interleukin‐1 beta; PI3K/AKT, phosphoinositide 3‐kinase/protein kinase B; PPAR, peroxisome proliferator‐activated receptor; SREBP1c, sterol regulatory element binding protein 1c; TNF‐α, tumor necrosis factor alpha.

Beyond its glycaemic and lipid effects, okra exerts broader systemic actions. Its flavonoids and vitamin C chelate iron, reducing generation of reactive oxygen species and hepatic oxidative stress (Khomsug et al., 2010). Polyphenolic compounds downregulate pro‐inflammatory cytokines, such as tumor necrosis factor‐alpha, interleukin (IL)‐6 and IL‐1β, while promoting anti‐inflammatory responses (Panighel et al., 2022). The mucilage fraction functions as a prebiotic, fostering the growth of beneficial gut bacteria (including *Bifidobacterium* and *Lactobacillus*) that improve gut barrier integrity and lower systemic inflammation (Zhou et al., 2024). Hormonal regulation through adiponectin and leptin further supports energy balance and insulin sensitivity (Esmaeilzadeh et al., 2020). Genetic polymorphisms, especially in glucose transporter type 4 (GLUT4), PPAR‐γ and SREBP1c, might contribute to interindividual variability in response to okra supplementation, and synergistic interactions with other dietary antioxidants might potentiate its metabolic benefits (Aligita et al., 2020; Gemede et al., 2018; Figure 3).

### 4.4 Clinical implications of findings

The findings of this meta‐analysis have important implications for the clinical management of T2DM. Evidence synthesized from RCTs conducted exclusively in diabetic populations indicates that okra supplementation exerts consistent benefits on glycaemic regulation, encompassing both short‐term measures, such as FBS and postprandial glucose, and longer‐term indicators, including HbA1c and HOMA‐IR. In addition, okra intake was associated with improvements in lipid parameters, particularly LDL‐C and TC, outcomes that are central to reducing cardiovascular risk in this population. Taken together, these effects suggest that okra might serve as a practical and low‐risk adjunct to conventional diabetes care, complementing established pharmacological and dietary strategies. In clinical settings, its use could be particularly relevant for patients who continue to demonstrate suboptimal glycaemic control or persistent dyslipidaemia despite adherence to standard treatment protocols. Subgroup findings further highlight its potential for individualized care; individuals with obesity appeared to derive greater benefit with respect to glycaemic indices, whereas lipid improvements were more evident in participants without obesity.

A key distinction of this review is its exclusive focus on patients with diabetes, in contrast to previous meta‐analyses that included mixed populations, such as individuals with prediabetes, obesity or metabolic syndrome. By restricting the analysis to diabetic cohorts, the present study provides a more disease‐specific and clinically actionable perspective. This narrower focus strengthens the translational value of the findings, offering an evidence base that is directly relevant to clinical decision‐making in diabetes management. Although heterogeneity in formulation, dosage and intervention duration across trials remains a limitation, the collective evidence supports the integration of okra supplementation as a complementary component within individualized management plans. Such an approach has the potential to enhance glycaemic control, improve lipid profiles and thereby contribute to lowering overall cardiovascular risk in patients with diabetes.

### 4.5 Strengths and limitations

This meta‐analysis offers several methodological strengths that advance the evidence base for okra supplementation in metabolic health. Although previous reviews, notably by Bahari et al. (2024), provided initial contributions, certain methodological limitations warranted re‐examination. We used an exhaustive, updated search strategy to 2025, systematically incorporating all eligible RCTs to ensure comprehensive evidence synthesis. Our analysis adopted methodological inclusivity by extracting and analysing all available outcomes, including those previously unreported, thereby minimizing selective reporting bias. We expanded outcome assessment beyond glycaemic and lipid markers to encompass liver function tests (ALT, AST and ALP), renal markers (creatinine), anthropometric parameters (BMI and waist circumference), 2 h postprandial glucose and inflammatory biomarkers (hs‐CRP), providing a holistic evaluation of the therapeutic effects okra's across metabolic, hepatic, cardiovascular and inflammatory domains. Rigorous data extraction procedures included standardized conversion methods for non‐normally distributed data and direct recalculation of effect sizes from raw data to ensure accuracy and clinical plausibility. We maintained strict inclusion criteria by excluding trials lacking analysable variance data. Additionally, we used advanced statistical approaches, including subgroup analyses stratified by multiple effect modifiers, meta‐regression to identify sources of heterogeneity, and non‐linear dose–response modelling to characterize optimal therapeutic thresholds, while implementing structured quality assessment using the Cochrane risk of bias tool and GRADE framework for certainty evaluation.

Beyond addressing these comparative gaps, our study demonstrates additional strengths. Its broad scope and rigorous methodology provide a comprehensive evaluation of okra supplementation across multiple cardiometabolic outcomes, including anthropometric measures, blood pressure, glycaemic indices, lipid profiles, and liver and renal function tests. By encompassing this wide range of end‐points, the study offers a more integrated understanding of the therapeutic potential of okra. Adherence to PRISMA guidelines ensured methodological transparency and reproducibility, and the inclusion of a substantial number of RCTs strengthened the validity of findings. Advanced statistical techniques, such as subgroup analyses, meta‐regression and non‐linear dose–response modelling, further enhanced robustness. These approaches helped to identify effect modifiers, including baseline BMI, age, dosage, intervention duration and formulation type, which are essential for tailoring therapeutic strategies. Use of the Cochrane risk of bias tool and the GRADE framework provided structured assessments of study quality and certainty, adding depth and clarity to the interpretation of results.

Another notable strength is the breadth and diversity of subgroup analyses. Stratification by health status (T2DM vs. IGT), supplementation approach (standardized products vs. lifestyle‐based use), formulation (capsules and tablets vs. natural or liquid forms) and co‐intervention strategy (monotherapy vs. combined treatments) revealed nuanced response patterns obscured in earlier reviews. For example, stronger effects observed in younger or obese participants and among those receiving liquid or natural formulations highlight the potential of okra as a personalized dietary intervention. The dose–response findings, which suggest an optimal threshold of ∼50 000 mg/day for glycaemic outcomes, represent another key contribution, offering a plausible explanation for discrepancies in prior literature.

Despite these strengths, several limitations must be acknowledged. Although 14 trials were included, many enrolled <100 participants, which limited statistical power and increased susceptibility to small‐study effects. Considerable heterogeneity was observed for outcomes such as FBS, HbA1c, TG and TC, complicating the interpretation of pooled estimates. Although random‐effects models and sensitivity analyses were applied, substantial residual heterogeneity suggests that differences in study populations, interventions and outcome definitions remain important sources of inconsistency. Moreover, some significant findings, including those for 2 h PPG, HbA1c and HOMA‐IR, lost significance in sensitivity analyses, underscoring the fragility in the evidence base.

Intervention protocols also varied widely. Okra supplementation was delivered in diverse forms (powder, extracts or capsules) at doses ranging from 80 to 80 000 mg/day and for durations of 2–24 weeks. The substantial variation in doses across studies, spanning three orders of magnitude, particularly impacts the generalizability of our findings, because it is unclear how these disparate regimens translate to standardized clinical applications or specific patient subgroups with varying metabolic needs. Such heterogeneity complicates comparisons and limits the generalizability of dose–response conclusions. Differences in plant parts used (pods, seeds or whole plant) are likely to have introduced further variability in phytochemical content and biological activity. Frequent use of co‐interventions, including pharmacological agents and dietary modifications, added another layer of confounding. The geographical concentration of trials (predominantly in Iran and a few other Asian countries) further restricts external validity, because cultural dietary practices, nutritional status and genetic backgrounds might influence responsiveness. The exclusion of non‐English publications raises the possibility of language bias.

The quality of evidence also warrants cautious interpretation. Nearly half of the included trials were assessed as having poor or fair methodological quality, and the overall certainty of evidence, as rated by the GRADE framework, was very low for most outcomes (low only for HbA1c and DBP). Short intervention durations, common across the included studies, restricted the evaluation of long‐term benefits, particularly regarding sustained cardiometabolic risk reduction or durability of effects. As a result, although the analysis provides meaningful insights into intermediate biomarkers, implications for hard clinical end‐points, such as cardiovascular events, diabetes complications or mortality, remain speculative.

### 4.6 Future directions

Although the present evidence supports the glycaemic and lipid‐lowering potential of okra supplementation, several critical questions remain unanswered. Most available trials are of short duration and involve relatively small cohorts, limiting the ability to draw firm conclusions regarding sustained efficacy, long‐term safety and outcomes such as cardiovascular events, diabetic complications or mortality. Large‐scale, multicentre RCTs with extended follow‐up are essential to confirm benefits and evaluate risks, particularly in individuals with multiple comorbidities. Variation in dosage, duration and formulation across existing studies further underscores the need to define optimal regimens. Emerging evidence suggests that dosage and preparation might influence outcomes differently: lower doses appear more effective for lipid regulation, whereas higher doses show greater impact on glycaemic control. Likewise, natural and liquid preparations have demonstrated stronger effects on FBS and insulin resistance, whereas capsule and tablet forms have yielded more favourable lipid responses. Comparative, dose‐ranging trials are therefore crucial to establish the most effective strategies for specific metabolic targets.

Patient‐related characteristics also warrant closer investigation. Differences in efficacy between diabetic and non‐diabetic individuals, across age groups and among BMI categories, highlight the importance of subgroup‐specific analyses. Broader inclusion of diverse populations, particularly beyond Iran and other Asian settings, will improve external validity. In addition, studies exploring genetic polymorphisms in key pathways, such as GLUT4, PPAR‐γ and SREBP1c, could clarify interindividual variability and inform personalized supplementation strategies.

Mechanistic understanding remains incomplete. Although preclinical work implicates inhibition of carbohydrate‐digesting enzymes, bile acid binding, modulation of PPAR and PI3K/Akt signalling, and attenuation of inflammation, clinical trials rarely integrate mechanistic end‐points. Future investigations should include biomarkers of insulin secretion, lipid metabolism, gut microbiota composition and inflammatory mediators to elucidate causal pathways. Advanced molecular biology and omics‐based approaches might also identify bioactive compounds within okra and their specific molecular targets. Potential interactions with commonly prescribed medications for diabetes, hypertension and dyslipidaemia remain underexplored. Systematic evaluation of drug–nutrient interactions is needed to ensure safe integration into clinical care. Likewise, studies assessing potential synergy with other natural compounds or conventional therapies could uncover new therapeutic opportunities. Expanding research to underrepresented populations, such as those with metabolic syndrome, gestational diabetes or autoimmune conditions, is also important. Beyond metabolic disorders, the putative anti‐cancer, hepatoprotective and renoprotective properties of okra merit rigorous clinical evaluation. Trials comparing whole fruit, extracts and isolated compounds could help to optimize delivery methods and guide the development of functional foods or nutraceuticals. Comprehensive safety data remain limited. Future RCTs should incorporate systematic adverse event reporting, long‐term tolerability assessments and cost‐effectiveness analyses. Such evidence would support clinicians, policy‐makers and insurers in determining the feasibility of integrating okra supplementation into standard care and dietary guidelines.

## 5 CONCLUSION

This meta‐analysis demonstrates that okra supplementation improves both glycaemic control and lipid regulation, underscoring its potential as a complementary nutritional strategy in diabetes management. The findings suggest that okra might help to address persistent dysglycaemia and dyslipidaemia despite standard therapies, with implications for clinical practice and public health. Although existing evidence is constrained by small and heterogeneous trials, these results provide a promising basis for future investigations aimed at defining optimal dosing, establishing long‐term safety and advancing personalized applications in the management of metabolic diseases.

## AUTHOR CONTRIBUTIONS

Conceptualization: Ali Jafari. Data curation: Ali Jafari, Helia Mardani, Mohammad Amin Karimi, Nooshin Enayati Soofi. Formal analysis: Ali Jafari, Ghazaleh Eslamian. Investigation: Ali Jafari, Helia Mardani, MohammadHossein Sahami Gilan, Helia Hemmat, Mohammad Amin Karimi, Ghazaleh Eslamian. Methodology: Ali Jafari, Ghazaleh Eslamian. Project administration: Ali Jafari, Ghazaleh Eslamian. Software: Ali Jafari, Helia Mardani, Ghazaleh Eslamian. Visualization: Fatemeh Shoja. Supervision: Ali Jafari, Ghazaleh Eslamian. Validation: Ali Jafari, Helia Mardani, MohammadHossein Sahami Gilan, Helia Hemmat, Nooshin Enayati Soofi, Fatemeh Shoja, Ghazaleh Eslamian. Writing—original draft: Ali Jafari, Helia Mardani, Mohammad Amin Karimi, MohammadHossein Sahami Gilan. Writing—review & editing: Ali Jafari, Helia Hemmat, Nooshin Enayati Soofi, Fatemeh Shoja, Ghazaleh Eslamian. All authors approved the final version of the manuscript and agree to be accountable for all aspects of the work in ensuring that questions related to the accuracy or integrity of any part of the work are appropriately investigated and resolved. All persons designated as authors qualify for authorship, and all those who qualify for authorship are listed.

## CONFLICT OF INTEREST

Ali Jafari holds an editorial position at *Systematic Reviews*. All other authors of this publication declare that they have no affiliations with, or involvement in, any organization or entity with a financial interest (including honoraria, educational grants, participation in speakers’ bureaus, memberships, employment, consultancies, stock ownership or other equity interests, expert testimony or patent‐licensing arrangements) or a non‐financial interest (such as personal or professional relationships, affiliations, knowledge or beliefs) relevant to the subject matter or materials discussed in this manuscript.

## FUNDING INFORMATION

None.

## Supporting information




**Supplementary Materials**: eph70214‐sup‐0001‐SuppMat.docx

## Data Availability

All relevant data are included in the manuscript and its supplementary materials. Moreover, the datasets analysed during the study are available from the corresponding author upon reasonable request.
