# Supplementary material for: Therapeutic potential of okra (Abelmoschus esculentus) in dysglycaemia and metabolic dysfunction: A systematic review and meta‐analysis across the diabetes spectrum
Source: Exp Physiol. 2026 Mar 13:10.1113/EP093293. Online ahead of print. doi: 10.1113/EP093293 (PMC13394657; doi:10.1113/EP093293)
Supplement: Supplementary file 1 — Supplementary Materials: eph70214‐sup‐0001‐SuppMat.docx [file EPH-9999-0-s001.docx]

**Therapeutic Potential of Okra (*****Abelmoschus esculentus*) in Dysglycemia and Metabolic Dysfunction: A Systematic Review and Meta-Analysis Across the Diabetes Spectrum**

Supplementary materials**:** Supplementary Tables 1-2, Supplementary Figure 1-12, and Supplementary References.

| **Supplementary Table 1.** Search strategy to find potential eligible randomised controlled trials (July 23, 2025) | |
| --- | --- |
| **Groups** | **Descriptors** |
| Intervention | Okra OR Okro OR "Abelmoschus esculentus" OR “Abelmoschus" OR “Hibiscus Esculentus" OR "Lady's Finger" OR Bamia OR Bhindi |
| Design | Intervention OR “Intervention Study” OR “Intervention Studies” OR “controlled trial” OR random* OR placebo OR “clinical trial” OR Trial OR “randomized controlled trial” OR “randomized clinical trial” OR RCT OR blinded OR “double blind” OR “double blinded” OR trial* OR “Pragmatic Clinical Trial” OR “Cross-Over Studies” OR “Cross-Over” OR “Cross-Over Study” OR parallel OR “parallel study” OR “parallel trial” |

**PubMed**

Number of localized studies: 28

|  | **Descriptors** | **Number of studies reached** |
| --- | --- | --- |
| #1 | ("Okra"[Title/Abstract] OR "Okro"[Title/Abstract] OR "Abelmoschus esculentus"[Title/Abstract] OR "Abelmoschus"[Title/Abstract] OR "Hibiscus Esculentus"[Title/Abstract] OR "Lady's Finger"[Title/Abstract] OR "Bamia"[Title/Abstract] OR "Bhindi"[Title/Abstract]) AND ("meta analysis"[Publication Type] OR "systematic review"[Filter])) OR "Abelmoschus"[MeSH Terms] | 584 |
| #2 | "Diabetes Mellitus"[MeSH Terms] OR "diabetes mellitus, type 2"[MeSH Terms] OR "diabetes mellitus, type 1"[MeSH Terms] OR "diabetes, gestational"[MeSH Terms] OR ("diabetes"[Title/Abstract] OR "Diabetes Mellitus"[Title/Abstract] OR "type 1 diabetes mellitus"[Title/Abstract] OR ("Diabetes Mellitus"[Title/Abstract] AND "type 1"[Title/Abstract]) OR "type 2 diabetes"[Title/Abstract] OR "T2DM"[Title/Abstract] OR "diabet*"[Title/Abstract] OR ("Diabetes Mellitus"[Title/Abstract] AND "type 2"[Title/Abstract]) OR "type 2 diabetes mellitus"[Title/Abstract] OR "gestational diabetes"[Title/Abstract] OR "gestational diabetes mellitus"[Title/Abstract] OR "GDM"[Title/Abstract] OR "pregnancy induced diabetes"[Title/Abstract] OR "gestational hyperglycemia"[Title/Abstract] OR "gestational glucose intolerance"[Title/Abstract] OR ("type 1 diabetes mellitus"[Title/Abstract] AND "pregnancy"[Title/Abstract]) OR ("type 1 diabetes"[Title/Abstract] AND "pregnancy"[Title/Abstract]) OR "pre-GDM"[Title/Abstract] OR "diabetes in pregnancy"[Title/Abstract]) | 946,281 |
| #3 | Intervention[Title/Abstract] OR "Intervention Study"[Title/Abstract] OR "Intervention Studies"[Title/Abstract] OR "controlled trial"[Title/Abstract] OR random*[Title/Abstract] OR placebo[Title/Abstract] OR "clinical trial"[Title/Abstract] OR Trial[Title/Abstract] OR "randomized controlled trial"[Title/Abstract] OR "randomized clinical trial"[Title/Abstract] OR RCT[Title/Abstract] OR blinded[Title/Abstract] OR "double blind"[Title/Abstract] OR "double blinded"[Title/Abstract] OR trial*[Title/Abstract] OR "Pragmatic Clinical Trial"[Title/Abstract] OR "Cross-Over Studies"[Title/Abstract] OR "Cross-Over"[Title/Abstract] OR "Cross-Over Study"[Title/Abstract] OR parallel[Title/Abstract] OR "parallel study"[Title/Abstract] OR "parallel trial"[Title/Abstract] OR "Clinical Trial" [Publication Type] OR "Randomized Controlled Trial" [Publication Type] OR "Controlled Clinical Trial" [Publication Type] OR "Random Allocation"[Mesh] OR "Randomized Controlled Trials as Topic"[Mesh] OR "Pragmatic Clinical Trial" [Publication Type] OR "Pragmatic Clinical Trials as Topic"[Mesh] OR "Double-Blind Method"[Mesh] OR "Single-Blind Method"[Mesh] OR "Cross-Over Studies"[Mesh] | 3,945,731 |
| #4 | #1 AND #2 AND #3 | 28 |

**Web of Science**

Number of localized studies: 66

|  | **Descriptors** | **Number of studies reached** |
| --- | --- | --- |
| #1 | **TS=(Okra OR Okro OR "Abelmoschus esculentus" OR “Abelmoschus" OR “Hibiscus Esculentus" OR "Lady's Finger" OR Bamia OR Bhindi)** | 5,035 |
| #2 | TS=(diabetes OR "Diabetes Mellitus" OR “type 1 diabetes mellitus” OR T1D* OR (“Diabetes Mellitus” AND Type 1) OR “type 2 diabetes” OR T2DM OR diabet* OR (“Diabetes Mellitus” AND Type 2) OR “type 2 diabetes mellitus” OR “gestational diabetes” OR “gestational diabetes mellitus” OR GDM OR “pregnancy induced diabetes” OR “gestational hyperglycemia” OR “gestational glucose intolerance” OR (“type 1 diabetes mellitus” AND pregnancy) OR (“type 1 diabetes” AND pregnancy) or “diabetes mellitus type 1 in pregnancy” or “diabetes type 1 in pregnancy” OR “type 2 diabetes in pregnancy” OR “type 2 diabetes mellitus in pregnancy” OR “pre-GDM” OR “diabetes in pregnancy”) | 1,111,996 |
| #3 | TS=(Intervention OR “Intervention Study” OR “Intervention Studies” OR “controlled trial” OR random* OR placebo OR “clinical trial” OR Trial OR “randomized controlled trial” OR “randomized clinical trial” OR RCT OR blinded OR “double blind” OR “double blinded” OR trial* OR “Pragmatic Clinical Trial” OR “Cross-Over Studies” OR “Cross-Over” OR “Cross-Over Study” OR parallel OR “parallel study” OR “parallel trial”) | 6,949,551 |
| #4 | #1 AND #2 AND #3 | 66 |

**Scopus**

Number of localized studies: 86

|  | **Descriptors** | **Number of studies reached** |
| --- | --- | --- |
| #1 | TITLE-ABS-KEY ( okra OR okro OR "Abelmoschus esculentus" OR "Abelmoschus" OR "Hibiscus Esculentus" OR "Lady's Finger" OR bamia OR bhindi ) | 6,598 |
| #2 | TITLE-ABS-KEY ( diabetes OR "Diabetes Mellitus" OR "type 1 diabetes mellitus" OR t1d* OR ( "Diabetes Mellitus" AND type 1 ) OR "type 2 diabetes" OR t2dm OR diabet* OR ( "Diabetes Mellitus" AND type 2 ) OR "type 2 diabetes mellitus" OR "gestational diabetes" OR "gestational diabetes mellitus" OR gdm OR "pregnancy induced diabetes" OR "gestational hyperglycemia" OR "gestational glucose intolerance" OR ( "type 1 diabetes mellitus" AND pregnancy ) OR ( "type 1 diabetes" AND pregnancy ) OR "diabetes mellitus type 1 in pregnancy" OR "diabetes type 1 in pregnancy" OR "type 2 diabetes in pregnancy" OR "type 2 diabetes mellitus in pregnancy" OR "pre-GDM" OR "diabetes in pregnancy" ) | 1,441,504 |
| #3 | TITLE-ABS-KEY (intervention OR "Intervention Study" OR "Intervention Studies" OR "controlled trial" OR random* OR placebo OR "clinical trial" OR trial OR "randomized controlled trial" OR "randomized clinical trial" OR rct OR blinded OR "double blind" OR "double blinded" OR trial* OR "Pragmatic Clinical Trial" OR "Cross-Over Studies" OR "Cross-Over" OR "Cross-Over Study" OR parallel OR "parallel study" OR "parallel trial" ) | 8,599,968 |
| #4 | #1 AND #2 AND #3 | 86 |

**Embase**

Number of localized studies: 141

|  | **Descriptors** | **Number of studies reached** |
| --- | --- | --- |
| #1 | okra:ti,ab,kw OR okro:ti,ab,kw OR 'abelmoschus esculentus':ti,ab,kw OR 'abelmoschus':ti,ab,kw OR 'hibiscus esculentus':ti,ab,kw OR bamia:ti,ab,kw OR bhindi:ti,ab,kw OR 'okra'/exp | 1,915 |
| #2 | diabetes:ti,ab,kw OR 'diabetes mellitus':ti,ab,kw OR 'type 1 diabetes mellitus':ti,ab,kw OR t1d*:ti,ab,kw OR ('diabetes mellitus':ti,ab,kw AND 'type 1':ti,ab,kw) OR 'type 2 diabetes':ti,ab,kw OR t2dm:ti,ab,kw OR diabet*:ti,ab,kw OR ('diabetes mellitus':ti,ab,kw AND 'type 2':ti,ab,kw) OR 'type 2 diabetes mellitus':ti,ab,kw OR 'gestational diabetes':ti,ab,kw OR 'gestational diabetes mellitus':ti,ab,kw OR gdm:ti,ab,kw OR 'pregnancy induced diabetes':ti,ab,kw OR 'gestational hyperglycemia':ti,ab,kw OR 'gestational glucose intolerance':ti,ab,kw OR ('type 1 diabetes mellitus':ti,ab,kw AND pregnancy:ti,ab,kw) OR ('type 1 diabetes':ti,ab,kw AND pregnancy:ti,ab,kw) OR 'diabetes mellitus type 1 in pregnancy':ti,ab,kw OR 'diabetes type 1 in pregnancy':ti,ab,kw OR 'type 2 diabetes in pregnancy':ti,ab,kw OR 'type 2 diabetes mellitus in pregnancy':ti,ab,kw OR 'pre-gdm':ti,ab,kw OR 'maternal diabetes mellitus':ti,ab,kw OR  'diabetes mellitus'/exp | 1,730,246 |
| #3 | 'randomized controlled trial'/exp OR 'randomized controlled trial (topic)'/exp OR 'pragmatic trial'/exp OR 'clinical trial'/exp OR 'clinical trial (topic)'/exp OR 'intervention study'/exp OR 'controlled study'/exp OR 'controlled clinical trial'/exp OR 'double blind procedure'/exp OR 'single blind procedure'/exp OR 'crossover procedure'/exp OR 'parallel design'/exp | 13,211,638 |
| #4 | #1 AND #2 AND #3 | 141 |

**Cochrane**

Number of localized studies: 34

Limits: TRIALS

|  | **Descriptors** | **Number of studies reached** |
| --- | --- | --- |
| #1 | **(Okra OR Okro OR "Abelmoschus esculentus" OR “Abelmoschus" OR “Hibiscus Esculentus" OR "Lady's Finger" OR Bamia OR Bhindi):ti,ab,kw** | 73 |
| #2 | (diabetes OR "Diabetes Mellitus" OR “type 1 diabetes mellitus” OR T1D* OR (“Diabetes Mellitus” AND Type 1) OR “type 2 diabetes” OR T2DM OR diabet* OR (“Diabetes Mellitus” AND Type 2) OR “type 2 diabetes mellitus” OR “gestational diabetes” OR “gestational diabetes mellitus” OR GDM OR “pregnancy induced diabetes” OR “gestational hyperglycemia” OR “gestational glucose intolerance” OR (“type 1 diabetes mellitus” AND pregnancy) OR (“type 1 diabetes” AND pregnancy) or “diabetes mellitus type 1 in pregnancy” or “diabetes type 1 in pregnancy” OR “type 2 diabetes in pregnancy” OR “type 2 diabetes mellitus in pregnancy” OR “pre-GDM” OR “diabetes in pregnancy” )**:ti,ab,kw** | 128,429 |
| #3 | #1 AND #2 | 34 |

| **Supplementary Table 2.** A summary of excluded articles after full text review | |
| --- | --- |
| **Author, Year (Ref.)** | **Reason** |
| Davis, 2012 (1) | Insufficient data |
| Taniguchi-Fukatsu, 2012 (2) | Not interested intervention (combined) |
| Labadnoy, 2017 (3) | Insufficient data |
| Shu-guo, 2018 (4) | Not interested intervention |
| ADAP, 2019 (5) | Not interested study design (quasi-experimental), limits of time |
| Haryati, 2019 (6) | Not interested study design (quasi-experimental) |
| Salarfard, 2019 (7) | Not interested outcome |
| Sarbini, 2019 (8) | Not interested intervention |
| Ziaee, 2019 (9) | Not access to full text |
| Nurseskasatmata, 2021(10) | Not interested study design |
| Zuniawati, 2021 (11) | Not interested study design (one group pre- post design) |
| Shobeiri, 2022 (12) | Not interested outcome |
| Zhao, 2022 (13) | Not interested outcome |
| Septiningtiyas, 2025 (14) | Not interested study design (quasi-experimental) |

**(a)**

**(b)**

**(c)**

**Supplementary Figure 1.** Forest plot of the effects of okra supplement on anthropometric measures (a: body mass index, b: waist circumference, c: weight)

**(a)**

**(b)**

**Supplementary Figure 2.** Forest plot of the effects of okra supplement on blood pressure (a: diastolic blood pressure, b: systolic blood pressure)

**(a)**

**(b)**

**(c)**

**(d)**

**(e)**

**Supplementary Figure 3.** Forest plot of the effects of okra supplement on glycemic profile (a: 2-hour postprandial glucose, b: fasting insulin, c: fasting blood sugar, d: hemoglobin a1c, e: homeostatic model assessment for insulin resistance)

**Supplementary Figure 4.** Forest plot of the effects of okra supplement on high-sensitivity C-reactive protein

**(a)**

**(b)**

**(c)**

**(d)**

**Supplementary Figure 5.** Forest plot of the effects of okra supplement on lipid profile (a: high-density lipoprotein cholesterol, b: low-density lipoprotein cholesterol, c: total cholesterol, d: triglycerides)

**(a)**

**(b)**

**(c)**

**Supplementary Figure 6.** Forest plot of the effects of okra supplement on liver function tests (a: alkaline phosphatase, b: alanine aminotransferase, c: aspartate aminotransferase)

**(a)**

**(b)**

**Supplementary Figure 7.** Forest plot of the effects of okra supplement on renal function tests (a: blood urea nitrogen, b: creatinine)


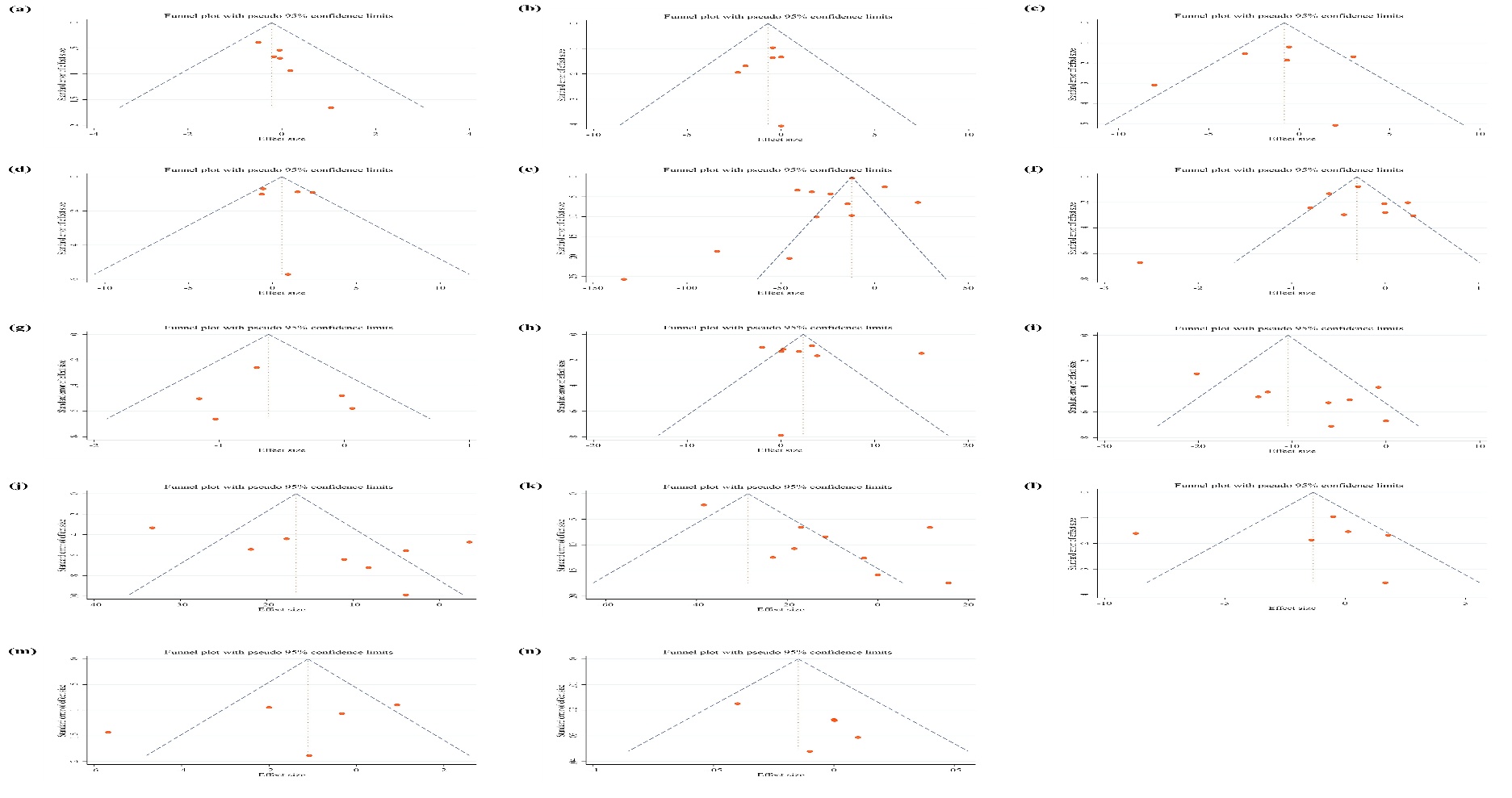


**Supplementary Figure 8.** Funnel plot assessing potential publication bias in the meta-analysis of okra supplementation on metabolic risk factors in diabetes (a: body mass index, b: diastolic blood pressure, c: systolic blood pressure, d: fasting insulin, e: fasting blood sugar, f: hemoglobin a1c, g: homeostatic model assessment for insulin resistance, h: high-density lipoprotein cholesterol, i: low-density lipoprotein cholesterol, j: total cholesterol, k: triglycerides, l: alanine aminotransferase, m: aspartate aminotransferase, n: creatinine)


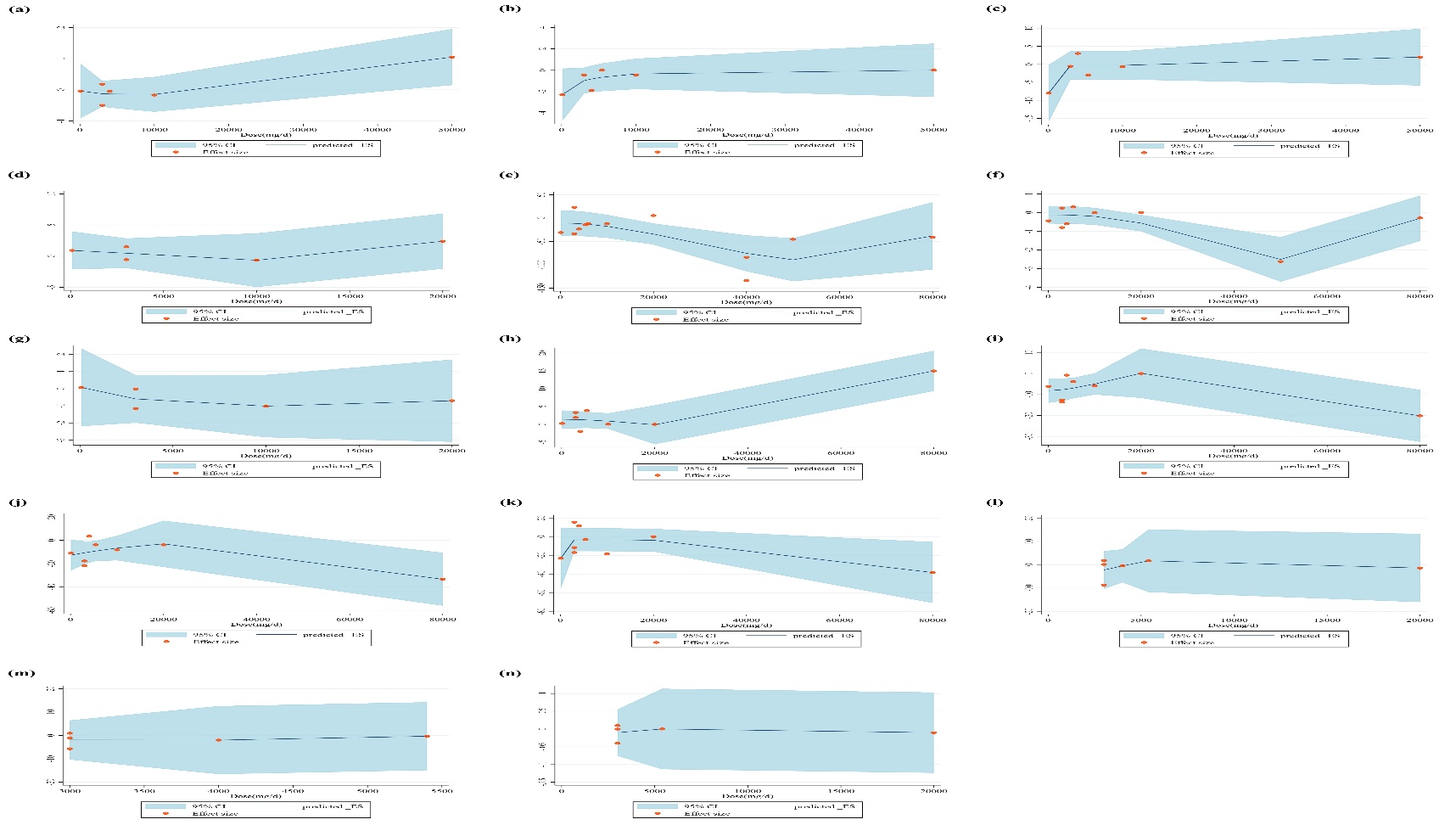


**Supplementary Figure 9.** Non-linear dose-response relations between okra dosage (mg/d) and metabolic risk factors in diabetes (a: body mass index, b: diastolic blood pressure, c: systolic blood pressure, d: fasting insulin, e: fasting blood sugar, f: hemoglobin a1c, g: homeostatic model assessment for insulin resistance, h: high-density lipoprotein cholesterol, i: low-density lipoprotein cholesterol, j: total cholesterol, k: triglycerides, l: alanine aminotransferase, m: aspartate aminotransferase, n: creatinine)


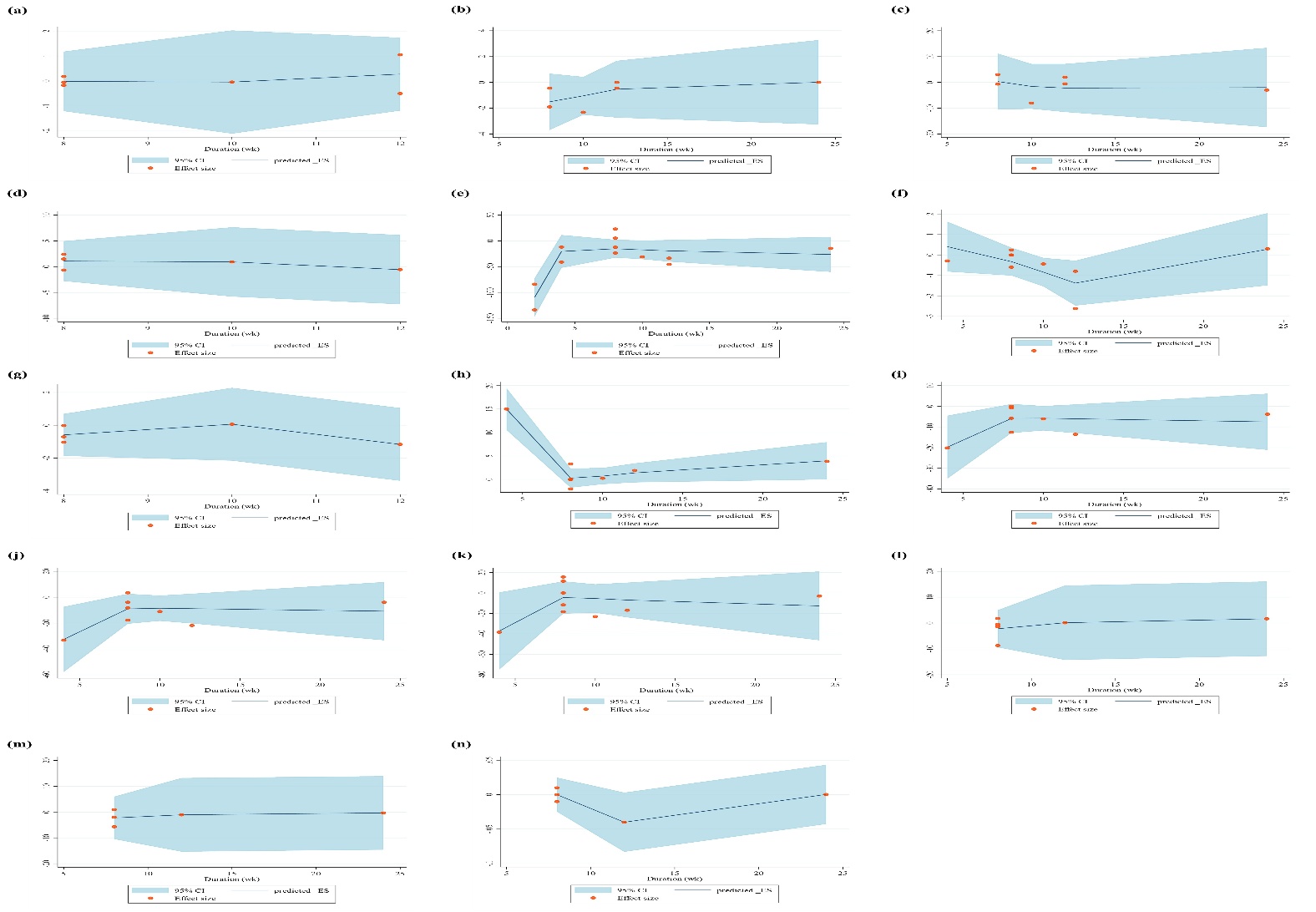


**Supplementary Figure 10.** Non-linear dose-response relations between duration of intervention and metabolic risk factors in diabetes (a: body mass index, b: diastolic blood pressure, c: systolic blood pressure, d: fasting insulin, e: fasting blood sugar, f: hemoglobin a1c, g: homeostatic model assessment for insulin resistance, h: high-density lipoprotein cholesterol, i: low-density lipoprotein cholesterol, j: total cholesterol, k: triglycerides, l: alanine aminotransferase, m: aspartate aminotransferase, n: creatinine)


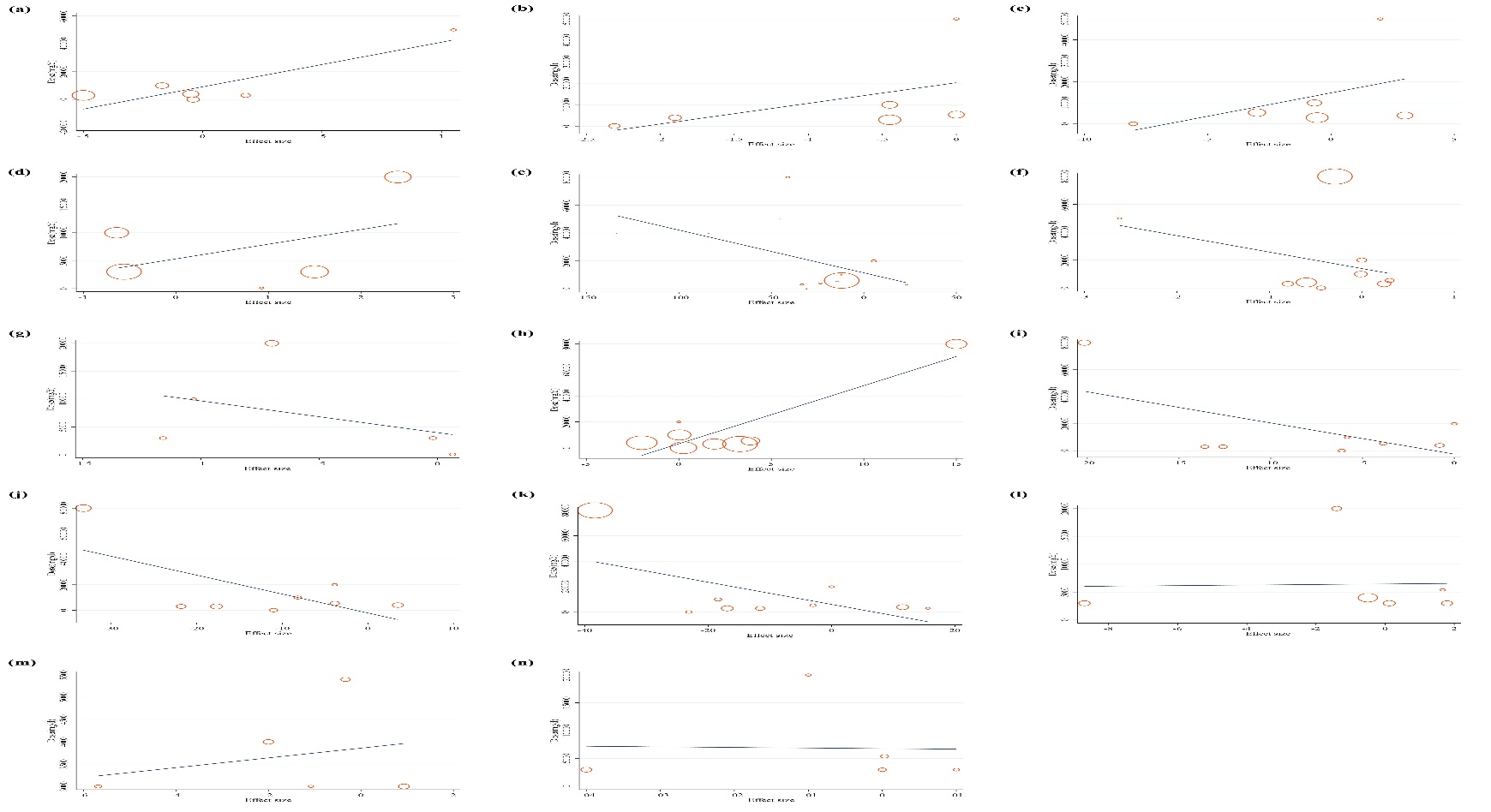


**Supplementary Figure 11.** Random-effects meta-regression plots of the association between okra dosage (mg/day) and metabolic risk factors in diabetes (a: body mass index, b: diastolic blood pressure, c: systolic blood pressure, d: fasting insulin, e: fasting blood sugar, f: hemoglobin a1c, g: homeostatic model assessment for insulin resistance, h: high-density lipoprotein cholesterol, i: low-density lipoprotein cholesterol, j: total cholesterol, k: triglycerides, l: alanine aminotransferase, m: aspartate aminotransferase, n: creatinine)


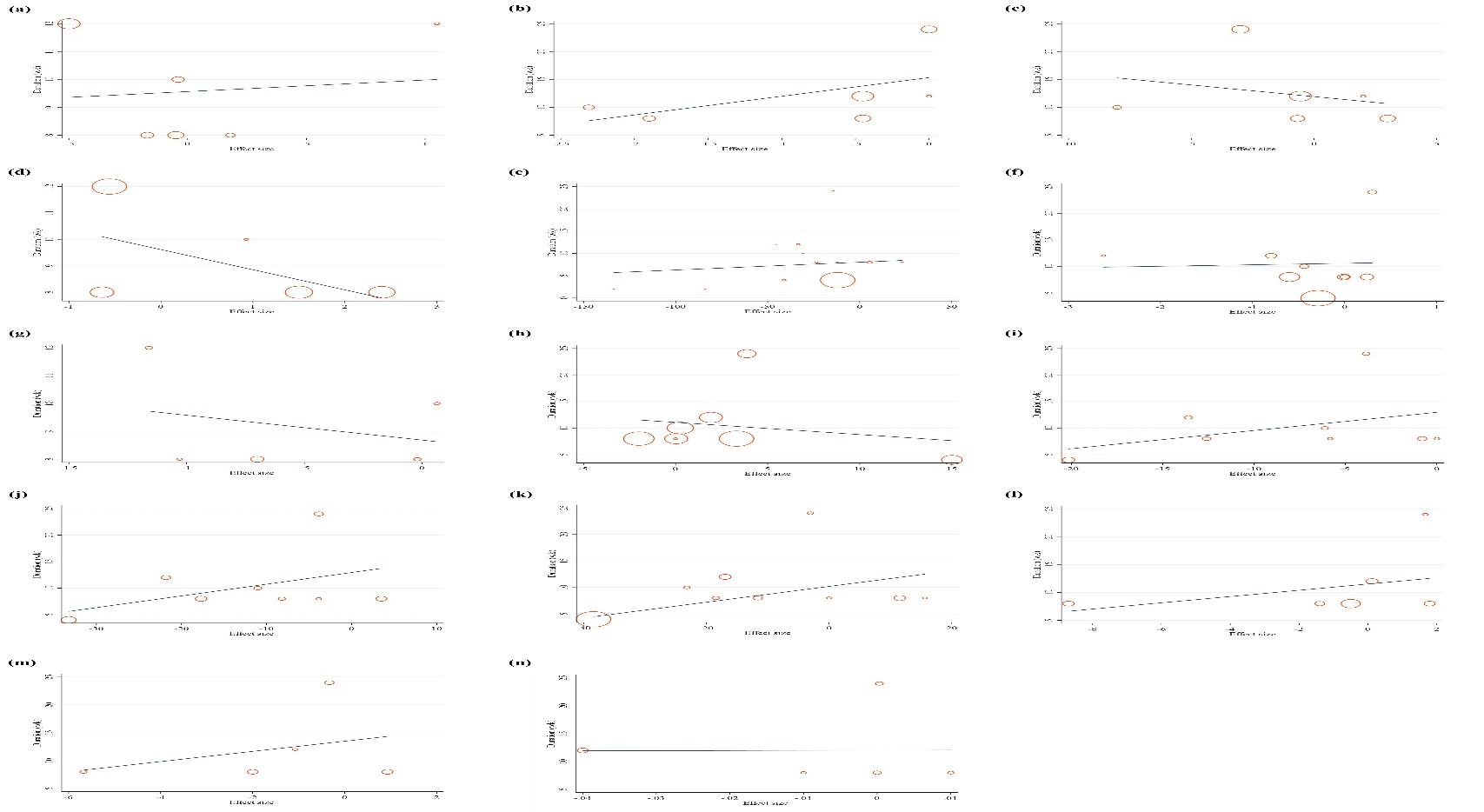


**Supplementary Figure 12.** Random-effects meta-regression plots of the association between duration of intervention and metabolic risk factors in diabetes (a: body mass index, b: diastolic blood pressure, c: systolic blood pressure, d: fasting insulin, e: fasting blood sugar, f: hemoglobin a1c, g: homeostatic model assessment for insulin resistance, h: high-density lipoprotein cholesterol, i: low-density lipoprotein cholesterol, j: total cholesterol, k: triglycerides, l: alanine aminotransferase, m: aspartate aminotransferase, n: creatinine)

**Supplemental** **References**

1. Davis S, D’Souza J. Effectiveness of Lady’s Finger Juice in the Control of Blood Sugar Among Type 2 Diabetes Mellitus Clients Aged 45-60 Years in Selected Areas of Mangalore. International Journal of Scientific Research. 2012;3:374-5.

2. Taniguchi-Fukatsu A, Yamanaka-Okumura H, Naniwa-Kuroki Y, Nishida Y, Yamamoto H, Taketani Y, et al. Natto and viscous vegetables in a Japanese-style breakfast improved insulin sensitivity, lipid metabolism and oxidative stress in overweight subjects with impaired glucose tolerance. British journal of nutrition. 2012;107(8):1184-91.

3. Labadnoy WA, Lalican MFL, Larrazabal DLB, Llamas MB, Zulueta JLA. The efficacy of okra (Abelmoschus esculentus) in decreasing blood sugar levels among patients with impaired fasting glucose in Antipolo City. UER. 2017:23.

4. Shu-guo S, Ji-chao Z, Meng Q. The effect of vitamin D 3 combined with okra capsule treatment on early protein uria in the patients with type 2 diabetic kidney disease. Journal of Clinical Nephrology. 2018;18(9):542-5.

5. ADAP CJL, T DIAZ CR, Victorino PLJ, MENDOZA MKH, MACAYAN YYC, editors. Hypoglycemic activity of okra (abelmoschus esculentus) in participants with impaired fasting glucose. 11th International Scholars Conference; 2019.

6. Haryati M, Rahmawati S. The effectiveness of okra fruit (Abelmoschus Esculentus) on fasting blood sugar and total cholesterol level in type 2 diabetes mellitus. Ann Trop Med Public Health. 2019;22:463-71.

7. Salarfard M, Abedian Z, Mazloum SR, Rakhshande H, Akhlaghi F. Comparison of adherence to the diet of women with gestational diabetes under diet therapy between the groups of with and without okra powder. The Iranian Journal of Obstetrics, Gynecology and Infertility. 2019;22(9):45-54.

8. Sarbini D, Huriyati E, Sadewa H, Wahyuningsih MSH. The effect of rosella (Hibiscus sabdariffa linn) on insulin resistance in patients with type 2 diabetes mellitus: A randomized clinical trial. 2019.

9. Ziaee M, Khorrami A, Ghassemi-Moghaddam H, Bahadori A. The effects of hydroalcholic extract of Hibiscus esculentus pods on glycemic control and lipid profile in type II diabetic patients: Randomised controlled clinical trial. 8th Natl Congr Med Plants. 2019:24-5.

10. Nurseskasatmata SE, Suharto IPS. THE EFFECT OF OKRA DECOCTION ON BLOOD SUGAR AND BODY WEIGHT IN DIABETIC CLIENTS. UNEJ e-Proceeding. 2020:131-6.

11. Zuniawati D, editor Activity Test of Infused Water Okra to Wards Reduction of Blood Sugar Levels in Type II Diabetes Mellitus Patients in Gondang Tulungagung Village. The 3 rd Joint International Conferences; 2021.

12. Shobeiri M, Rad AHE, Sheikholeslami Z, Zenozian MS, Saeidiasl MR. Formulation if functional cake with quinoa and okra flour and investigated the effect on blood sugar in diabetic patients. 2022.

13. Zhao J, Tostivint I, Xu L, Huang J, Gambotti L, Boffa J-J, et al. Efficacy of combined abelmoschus manihot and irbesartan for reduction of albuminuria in patients with type 2 diabetes and diabetic kidney disease: a multicenter randomized double-blind parallel controlled clinical trial. Diabetes care. 2022;45(7):e113-e5.

14. Septiningtiyas AD, Anggraini DA. PENGARUH PEMBERIAN JUS SARI BUAH OKRA (ABELMOSCHUS ESCULENTUS) TERHADAP PENURUNAN KADAR GULA DARAH PADA LANSIA PENDERITA DIABETES MELLITUS. Enfermeria Ciencia. 2025;3(1):32-43.
